# Artificial intelligence-enabled electrocardiography contributes to hyperthyroidism detection and outcome prediction

**DOI:** 10.1038/s43856-024-00472-4

**Published:** 2024-03-12

**Authors:** Chin Lin, Feng-Chih Kuo, Tom Chau, Jui-Hu Shih, Chin-Sheng Lin, Chien-Chou Chen, Chia-Cheng Lee, Shih-Hua Lin

**Affiliations:** 1 School of Medicine, National Defense Medical Center, Taipei, Taiwan ROC; 2 Graduate Institute of Aerospace and Undersea Medicine, National Defense Medical Center, Taipei, Taiwan ROC; 3 Division of Endocrinology and Metabolism, Department of Internal Medicine, Tri-Service General Hospital, National Defense Medical Center, Taipei, Taiwan ROC; 4 Department of Medicine, Providence St. Vincent Medical Center, Portland, OR USA; 5 Department of Pharmacy Practice, Tri-Service General Hospital, Taipei, Taiwan ROC; 6 School of Pharmacy, National Defense Medical Center, Taipei, Taiwan ROC; 7 Division of Cardiology, Department of Internal Medicine, Tri-Service General Hospital, National Defense Medical Center, Taipei, Taiwan ROC; 8 Division of Nephrology, Department of Medicine, Tri-Service General Hospital, National Defense Medical Center, Taipei, Taiwan ROC; 9 Department of Medical Informatics, Tri-Service General Hospital, National Defense Medical Center, Taipei, Taiwan ROC; 10 Division of Colorectal Surgery, Department of Surgery, Tri-Service General Hospital, National Defense Medical Center, Taipei, Taiwan ROC

**Keywords:** Thyroid diseases, Diagnostic markers, Prognostic markers

## Abstract

**Background:**

Hyperthyroidism is frequently under-recognized and leads to heart failure and mortality. Timely identification of high-risk patients is a prerequisite to effective antithyroid therapy. Since the heart is very sensitive to hyperthyroidism and its electrical signature can be demonstrated by electrocardiography, we developed an artificial intelligence model to detect hyperthyroidism by electrocardiography and examined its potential for outcome prediction.

**Methods:**

The deep learning model was trained using a large dataset of 47,245 electrocardiograms from 33,246 patients at an academic medical center. Patients were included if electrocardiograms and measurements of serum thyroid-stimulating hormone were available that had been obtained within a three day period. Serum thyroid-stimulating hormone and free thyroxine were used to define overt and subclinical hyperthyroidism. We tested the model internally using 14,420 patients and externally using two additional test sets comprising 11,498 and 596 patients, respectively.

**Results:**

The performance of the deep learning model achieves areas under the receiver operating characteristic curves (AUCs) of 0.725–0.761 for hyperthyroidism detection, AUCs of 0.867–0.876 for overt hyperthyroidism, and AUC of 0.631–0.701 for subclinical hyperthyroidism, superior to a traditional features-based machine learning model. Patients identified as hyperthyroidism-positive by the deep learning model have a significantly higher risk (1.97–2.94 fold) of all-cause mortality and new-onset heart failure compared to hyperthyroidism-negative patients. This cardiovascular disease stratification is particularly pronounced in subclinical hyperthyroidism, surpassing that observed in overt hyperthyroidism.

**Conclusions:**

An innovative algorithm effectively identifies overt and subclinical hyperthyroidism and contributes to cardiovascular risk assessment.

## Plain language summary

Hyperthyroidism occurs when the thyroid gland produces too much hormone and can cause various symptoms including faster heartbeat, weight loss, and nervousness. Diagnosis is often missed, which can lead to heart problems and even death. Measurements of the heart’s electrical activity can be obtained using Electrocardiograms (ECGs). We made a computational model that can detect hyperthyroidism from ECGs. Our model was better able to identify people with hyperthyroidism than currently available methods, especially the more severe forms of the condition. If future work demonstrates our model is safe and accurate, it could potentially be used to detect hyperthyroidism sooner, enabling faster treatment and improved health of people with hyperthyroidism.

## Abstract (web summary)

Lin et al. develop an artificial intelligence model capable of using electrocardiograms (ECGs) to predict hyperthyroidism. This predictive model performs well at detecting overt hyperthyroidism and can also be used to assess the risk of mortality and heart failure, independent of laboratory based thyroid tests.

## Introduction

Hyperthyroidism (HT) is a common endocrine disorder with a prevalence of 0.8–1.3% and increases with age^1^. It is associated with increased morbidity, all-cause, and cardiovascular mortality if untreated^1^. Heart failure (HF) is the leading cause of increased cardiovascular mortality in HT^2^. The diagnosis of HT can be made by measuring serum thyroid-stimulating hormone (TSH), which has the highest sensitivity and specificity for evaluating suspected thyrotoxicosis among laboratory tests^3^. However, the symptoms of HT can mimic other illnesses, making early diagnosis difficult. Patients with HT are frequently underrecognized, and almost two-thirds of them still need to undergo appropriate laboratory evaluation^4^. Furthermore, laboratory-based thyroid function tests often require longer turnaround times. Therefore, tools to boost the timely diagnosis of HT are sorely needed.

Cardiovascular manifestations are among the most critical effects of thyroid hormone^5^. The electrocardiogram (ECG) is an inexpensive and non-invasive tool to characterize cardiac changes, popularly applied in clinical practice. Well-known ECG findings associated with HT include sinus tachycardia, increased QRS voltage, and atrial fibrillation^6^. Recently, artificial intelligence (AI) techniques based on deep learning models (DLMs)^7^ have been shown to achieve human-level performance and effectively detect cardiac and non-cardiac disorders affecting the heart using large, annotated ECG datasets^8,9^. As mentioned above, the heart is very sensitive to hyperthyroidism, and its electrical signature can be detected by non-invasive electrocardiography (ECG). A recent study has developed a DLM to detect overt HT with area under the receiver operating characteristic (ROC) curves (AUCs) of >0.88 using 12-lead ECG^10^. Although AI-enabled ECG (AI-ECG) systems have been shown to identify previvors of cardiovascular diseases (CVD)^9^, their prognostic value as independent predictors for HT-related cardiovascular disease has yet to be investigated.

In this retrospective cohort study, we aim to develop an artificial intelligence-enabled ECG (AI-ECG) system with internal and external validation to assess its diagnostic accuracy and outcome prediction in HT. The AI-ECG system detects overt HT with AUCs of >0.86 in the test sets of three different hospitals. AI-ECG suggested HT (positive AI-ECG) also predicts adverse outcomes such as all-cause mortality and new-onset HF in patients with HT and non-HT. Such digital augmentation can be used for opportunistic clinical screening to identify high-risk patients warranting further thyroid testing.

## Methods

### Ethics statement

This retrospective study was approved by the institutional review board of Tri-Service General Hospital, Taipei, Taiwan (IRB No. C202105049). The need for individual consent from patients was waived because data were collected retrospectively in anonymized files and encrypted from the hospital to the data controller.

### Data source and definition

This study included three separate institutions affiliated with a Taiwanese hospital system. An academic medical center in Neihu district, Taipei City, denoted as Hospital A, provided research data from January 2010 to February 2022. A community hospital located in Zhongzheng district, Taipei City, denoted as Hospital B, provided an external test set from May 2011 to February 2022; patients who ever visited Hospital A were excluded. We also collected data from a local hospital on Penghu Island, an isolated island off the main island of Taiwan, denoted as Hospital C, and outpatient department data from January 2021 to February 2022.

All 12-lead ECGs were recorded using a Philips system®, which yielded 5000 voltage–time data points for each lead, up to 35 ECG patterns, and 8 ECG measurements. Serum TSH determination was based on radioimmunoassay with a lower detection limit of 0.03 μIU/mL (Diagnostic Products Corporation, Los Angeles, CA, USA). This study defined a TSH of ≤0.50 μIU/mL as HT. We also collected the nearest fT4 within three days for each HT record; serum fT4 ≥ 1.78 ng/dL was defined as overt HT^11^. In addition to overt and subclinical HT determined by fT4 levels, we also defined TSH levels ≤0.05 μIU/mL as severe HT. Methimazole and propylthiouracil were the only available antithyroid drugs (ATDs) at these hospitals. The use of ATD was also recorded, and the first dispense date of a period of at least two months denoted the start of ATD therapy. Short-term usage of less than two months was excluded.

Patient characteristics were collected from each hospital’s information system, and their medical history prior to the index date was identified using ICD codes, which included the following: hyperthyroidism (HT, ICD-9 code 242.9 and ICD-10 codes E05.x), diabetes mellitus (DM, ICD-9 codes 250.x and ICD-10 codes E11.x), hypertension (HTN, ICD-9 codes 401.x to 404.x and ICD-10 codes I10.x to I16.x), hyperlipidemia (HLP, ICD-9 codes 272.x and ICD-10 codes E78.x), chronic kidney disease (CKD, ICD-9 codes 585.x and ICD-10 codes N18.x), acute myocardial infarction (AMI, ICD-9 codes 410.x and ICD-10 codes I21.x), stroke (STK, ICD-9 codes 430.x to 438.x and ICD-10 codes I60.x to I63.x), coronary artery disease (CAD, ICD-9 codes 410.x to 414.x, and 429.2, and ICD-10 codes I20.x to I25.x), heart failure (HF, ICD-9 codes 428.x, 398.91, and 402.x1, and ICD-10 codes I50.x), atrial fibrillation (Afib, ICD-9 code 427.31 and ICD-10 codes I48.x), and chronic obstructive pulmonary disease (COPD, ICD-9 codes 490.x to 496.x and ICD-10 codes J44.9). Additionally, we reviewed records of laboratory tests and transthoracic echocardiography for patients with HT and HF, identifying those with abnormal records from either source.

We also gathered traditional ECG features from the Philips system®. The Philips system® provided an automated analysis for each ECG, resulting in 35 ECG patterns and 8 ECG measurements extracted from XML documents. These 8 ECG measurements encompassed heart rate, PR interval, QRS duration, QT interval, corrected QT interval, P wave axis, RS wave axis, and T wave axis. The data for these eight variables were 91–100% complete. For missing values, we performed imputation using multiple imputations via chained equations, employing the R package “mice” version 3.5.0. The 35 ECG patterns were derived from statements generated by the Philips system® for each ECG. Each ECG underwent analysis by the Philips system®, which produced 3-6 statement codes. The 35 ECG patterns included: sinus rhythm (statements code: SR; ST; TWRV; SB), sinus arrhythmia (statements code: SA; SAB; SAT), sinus pause (statements code: SP; SARSV; SARA), ectopic atrial rhythm (statements code: EAR; EAB; EAT; SEAR; SEAB; SEAT), junctional rhythm (statements code: JER; JRA; JT; SDJ), pacemaker rhythm (statements code: PMR; WPACE; PSAR; NFAD; NFRA), early precordial R/S transition (statements code: ET), ST elevation (statements code: STE; MSTEL; BSTEAL; EREPOL; STELVH; MSTEAL; CINJI; BSTEA; MSTEI; CINJA; MSTEA; CINJL; MSTED; BSTEI; BSTE; PINJA; PINJL; PINJAL; CINJAL; SD0IN; BSTEL), ST depression (statements code: STD; SD1AL; SDJ; SDPRR; SD1IN; SD0DI; SD2AN; SD2WI; SD1AN; SD0AN; SDM; SD2IN; SD2AL; SD0AL; SD0LA; SD1LA; SD2NS; SD1DI), abnormal T wave (statements code: ATW; T1LA; T0IN; T0LA; T1LA; T1AN; T1IN; T0AN; T0DI; T1DI; T0NS; TTW10; T1AL; T3AL; T3LA; T6AL; T3WI; T3IN; T3AN; T0AL), abnormal Q wave (statements code: AQW; LATQ; INFQ; IMI3; ASQLVH; AMI57; AQLVH; PQIN; IMI18; PIMI; AMI17; AMI48; LMI10; ANTQ; LMI28), RSR’ wave (statements code: RSRW; RSR1; ETRSR1), low voltage (statements code: LVOL; LVOLFB; LVOLF; LVOLP; LVORAD; LVOLT), left axis deviation (statements code: LAD; AXL; CAFBII; NIVCDL), right axis deviation (statements code: RAD; AXR; LVORAD), left ventricular hypertrophy (statements code: LVH; LVH1; LVHVP; LVHSR; LVHR56; LVHV; LVHREP; STELVH; LVHCNP; LVHPRE; LVHSR; LVHCNV; ASQLVH; LVHR56; LVHCO; LVHV; AQLVH; TIALVH; LVHCOL; AMI57; AMI17; AMI48; LMI28), right ventricular hypertrophy (statements code: RVH; PRVH; CRVH; RVHR; PRVHR; CRVHR), left atrial enlargement (statements code: LAE; PLAE; CLAE; LAECB), right atrial enlargement (statements code: RAE; LAECB; BAE; RAECB; CRAE), left atrium abnormality (statements code: LAA; CRAA; PLAA), right atrium abnormality (statements code: RAA; CRAA; PRAA), nonspecific intraventricular conduction delay (statements code: NIVCD; BIVCD; IVCDP; LVHCO; NIVCDL), left fascicular block (statements code: LFB; LAFB; LPFB; CLAFB; RLAFB; CAFBII; IRAFB; RLPFB), right bundle branch block (statements code: RBBB; IRBBB; RLAFB; IRAFB; RLPFB; ARBBB), left bundle branch block (statements code: LBBB; ILBBB), first degree AV block (statements code: 1AVB), second degree AV block (statements code: 2AVB; AFL2; AFLT2; AFLT3; AFLT4; WENCK; MOBII), complete degree AV block (statements code: CAVB; 3AVB; 3AVBFF; 3AVBIR), atrial fibrillation (statements code: AFIB; AFIB0; 3AVBFF; VPACCF; VPACEF; FLFIB; AFIBT; AVDPCF), atrial flutter (statements code: AFLT; AFL2; AFLT2; AFLT3; AFLT4; 3AVBFF; AFLTV; FLFIB; VPACCF; VPACEF), supraventricular tachycardia (statements code: SVT), WPW syndrome (statements code: VPE), ventricular tachycardia (statements code: RVPC), and atrial premature complex (statements code: APC; MAPC; APCPR), ventricular premature complex (statements code: VPC; PVPC; APCPR; VBIG; MVPC; VTRI; MFVPC; IVPC).

### Training, validation, and test sets

**Fig. 1:**
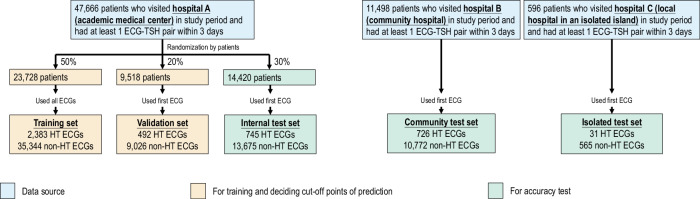
Study flow chart and dataset generation summary. This schematic illustrates the process of creating and analyzing the dataset to ensure a robust and reliable dataset for training, validation, and testing of the network. Each patient’s data was exclusively used within their assigned dataset to prevent cross-contamination between the training, validation, and test sets. The sky blue represents the data source level; orange signifies data used for training and determining cutoff points for prediction; while bluish green indicates data used for accuracy testing. Further details regarding the usage of each dataset are provided in the Methods section. HT hyperthyroidism.

Figure 1 summarizes the data set generation process in this study. All 12-lead ECGs recorded on patients who had also received at least one serum TSH test within three days before or after the ECG were used. Hospitals A, B, and C included 47,666, 11,498, and 596 eligible patients. Hyperthyroidism was defined as a TSH ≤ 0.50 μIU/mL. The patients from Hospital A were randomly partitioned for various uses. 50% of patients and all their ECG-TSH pairs comprised the DLM training set (HT: 2,383 ECGs and non-HT: 35,344 ECGs), and 20% of patients and their first ECG-TSH pair made up the validation set (*n*  =  492 and 9,026 for HT and non-HT ECGs). The remaining 30% of patients and their first ECG (*n*  =  745 and 13,675 for HT and non-HT ECGs) were reserved for internal accuracy testing. External validation of the model used unique data pairs obtained from Hospitals B and C. We only used each patient’s first ECG to avoid patient dependency. The community test set included 726 HT ECGs and 10,772 non-HT ECGs from Hospital B, and the isolated test set had 31 HT ECGs and 565 non-HT ECGs from Hospital C. Since the definition of an ECG-TSH pair was within three days of each other, patients may have had an ECG or TSH test before the first ECG-TSH pair, for instance, if they had a history of thyroid disease or ATD usage.

### Outcome measurement

The outcomes of interest included all-cause mortality and new-onset HF. We tracked each patient from their corresponding index date, which in the internal and community test sets was defined as the date of the ECG examination. Moreover, data for non-event visits were censored at the patient’s last known hospital alive encounter to limit bias from incomplete records. The endpoint of this study was set as Feb 28, 2022. Considering the first case in this study was collected in 2010, the maximum follow-up period was over ten years.

The electronic medical record captured patient status (dead/alive) for all-cause mortality. Although patients may have died at an outside hospital with a separate electronic medical record, we believe the prevalence is low as a previous study of readmissions in Taiwan using the government’s National Health Insurance Research Database found that only 0.16% of readmissions occurred at a different hospital^12^. Moreover, we ensured the censored patients were alive at the last known hospital encounter.

For new-onset HF, we excluded patients with a history of HF. The definition of HF included ICD codes (ICD-9 codes 428.x, 398.91, and 402.x1, and ICD-10 codes I50.x) and a transthoracic echo (TTE) report with an ejection fraction ≤35%. Since new-onset HF often requires hospitalization, we also estimated our incomplete data rate to be low based on patient loyalty to their hospital in Taiwan, as referenced above.

### Deep learning model and machine learning model

The DLM architecture, incorporating an attention mechanism, was employed to estimate the probability of HT, as per our prior study^13–15^. Supplementary Fig. 1A illustrates the structure of our DLM. Each ECG was recorded in the standard 12 leads, resulting in sequences of 5000 numbers, from which we generated a 5000 × 12 matrix. The input format for this architecture is a 4096 × 12 matrix. The creation of the input matrix is depicted in Supplementary Fig. 1B. We randomly selected a 4096-length sequence for input during the training phase. During the inference stage, we utilized two overlapping sequence lengths of 4096 justified to the beginning and end to generate predictions, which were then averaged to yield the final prediction.

We defined a “residual module” as a neural combination with a constant ‘k,’ structured as follows: (1) a 1 × 1 convolution layer with ‘k/4’ filters to reduce data dimensions; (2) a batch normalization layer for normalization; (3) a rectified linear unit (ReLU) layer for non-linearity; (4) a 3 × 1 convolution layer with ‘k/4’ filters to extract features; (5) a batch normalization layer for normalization; (6) a ReLU layer for non-linearity; (7) a 3 × 1 convolution layer with ‘4k’ filters to further extract features; (8) a 1 × 1 convolution layer with ‘k’ filters to restore the feature shape; (9) another batch normalization layer for normalization; (10) a ReLU layer for non-linearity; and (11) a squeeze-and-excitation (SE) module for feature weighting. The SE module was defined as follows: (1) an average global pooling layer; (2) a fully-connected layer with ‘k/r’ neurons; and (3) another fully-connected layer with ‘k’ neurons, where ‘r’ was consistently set to 8 in all experiments. Each residual module had a shortcut connection, directly connecting to all subsequent layers. When the size of feature maps changed, concatenation could not be performed, so our architecture used a “pool module” for down-sampling. This module included similar concatenated layers as the residual module but with a stride change in the 3 × 1 convolution layer (from 1 × 1 to 2 × 1). An average pooling layer with a 2 × 1 kernel size and stride was used for down-sampling, and the input data were integrated through the concatenated function.

The initial data underwent processing through a batch normalization layer, followed by an 11 × 1 convolution layer with a 2 × 1 stride and 16 filters, another batch normalization layer, a ReLU layer, and a pool module. Subsequently, the data passed through a series of residual and pool modules, resulting in a 32 × 12 × 1024 array. A global pooling layer was followed by the last residual module. This output was divided into 12 lead-specific feature maps, each with 1024 features. These feature maps underwent processing through a fully connected layer with one neuron to generate lead-specific predictions. A sigmoid function was applied to calculate the probability of HT for each lead. We designed an attention mechanism based on a hierarchical attention network to concatenate these blocks, thereby enhancing the interpretive power of the DLM. The attention module consisted of a fully connected layer with eight neurons, followed by a batch normalization layer, a ReLU layer, and a fully connected layer with one neuron to generate weights for each lead. Attention scores were calculated for each ECG lead and standardized by the last linear output layer. These standardized attention scores were used to weigh the 12 ECG lead outputs through simple multiplication. The 12 weighted outputs were summed and processed through a prediction module to produce the final prediction value.

We trained these DLMs with a batch size of 32 and initiated the learning rate at 0.001 using the Adam optimizer with standard parameters (β_1_ = 0.9 and β_2_ = 0.999). To ensure adequate recognition of HT cases, we implemented an oversampling process. For each batch, we sampled 16 cases from the pool of 2869 HT ECGs and 16 cases from the more extensive pool of 62,485 non-HT ECGs in the training set. The learning rate was reduced by a factor of 10 whenever the loss on the validation set reached a plateau after an epoch. To prevent the networks from overfitting, we employed early stopping. This involved saving the network after each epoch and selecting the saved DLMs that exhibited the lowest loss on the validation set. In this study, the only regularization method employed to prevent overfitting was L2 regularization, with a coefficient set at 10^−4^.

We also trained an XGB classifier using the same training, validation, and test sets as the DLM above to compare HT detection using deep learning and machine learning methods. The XGB classifier utilized all patient characteristics and ECG features. The training was conducted using the R package xgboost version 0.71.2, and all default prediction parameters were used. Additionally, we generated new predictions by combining the DLM’s predictions with patient characteristics using the XGB classifier. These predictions were used to evaluate and compare accuracy between the two approaches.

### Statistics and reproducibility

The patient characteristics are presented as means and standard deviations or percentages where appropriate. The Student t-test, analysis of variance (ANOVA), and chi-square tests were used for hypothesis tests. ROC curve and AUC were used to measure DLM accuracy. We also used multivariable Cox proportional hazard models to analyze the relationship between AI-ECG prediction and outcomes of interest. Hazard ratios (HRs) were used for comparison, and Kaplan–Meier curve analysis was used to calculate the cumulative incidence over time. The statistical analysis was conducted using the software environment R version 3.4.4 with a significance level of *p* < 0.05 and 95% confidence intervals (95% CIs).

### Reporting summary

Further information on research design is available in the Nature Portfolio Reporting Summary linked to this article.

## Results

### Patient characteristics

This study included adult patients (≥18 years old) who had undergone 12-lead ECG recordings and received at least one serum TSH test within 3-days before or after the ECG. The patients’ characteristics in the training, validation, and internal test sets are shown in Supplementary Table 1. The varying baseline characteristics between patients from three geographically distinct hospitals (Supplementary Table 2) allowed us to test the resiliency of our approach outside the derivation cohort. The eligible patient population from Hospitals A, B, and C comprised 47,666, 11,498, and 596 patients, respectively. The ECG-TSH pairs of patients were primarily obtained from outpatient settings (68.5% in Hospital A; 61.6% in Hospital B; 100.0% in Hospital C), while the remaining pairs were roughly evenly divided between the emergency department (16.2% in Hospital A; 18.2% in Hospital B) and inpatients (15.2% in Hospital A; 20.1% in Hospital B). The patient characteristics of HT and non-HT cases in the test sets are shown in Supplementary Table 3. Patients with HT were prone to be female and had more HTN. Their lesser other comorbidities might be less due to younger age. Patients without HT had a higher proportion of ECG-TSH pairs within one day. The proportion of overt HT in the internal, community, and isolated test sets were 37.7%, 41.8%, and 51.6% of HT, respectively, and 16.0–32.3% had a prior history of HT, and 6.8–12.5% had a history of ATD. Supplementary Table 4 shows the detailed comparison between overt and subclinical HT. Patients with overt HT were mainly younger outpatients with fewer comorbidities, which conversely implies the complexity of the subclinical HT population.

### The diagnostic value of AI-ECG for HT

**Fig. 2:**
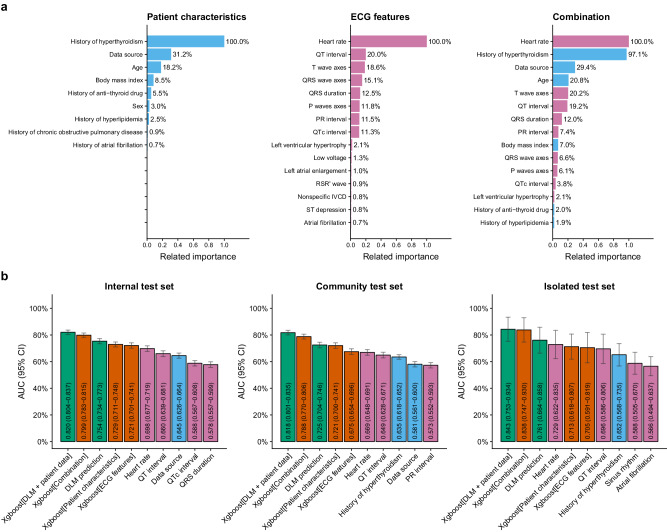
A comparison between AI-ECG and all available data. **a** Components of traditional machine learning models for detecting hyperthyroidism (HT). We trained three xgboost models to predict HT using patient characteristics, ECG features, and a combination of both. The sky-blue bars represent patient characteristics, while the reddish-purple bars represent ECG features. **b** The area under ROC curve (AUC) of all available data on HT. This includes the DLM using ECG waveform data only and the DLM combined with patient characteristics. The sky-blue and reddish-purple bars represent the results of predictions using individual patient characteristics and ECG features, respectively. The vermillion bars represent predictions integrating features from xgboost, and the bluish-green bars represent predictions, including those from DLM. The error bars are the 95% confidence intervals (CI) of each AUC.

We initially compared the performance of DLM using raw ECG signals with traditional feature-based machine learning models (MLMs) for HT detection. Figure 2A illustrates the composition of these MLMs, with patient characteristics, particularly history of HT and data source being the most important factors. The most crucial ECG feature was the heart rate. Figure 2B compares the AUC of these parameters, MLMs, and DLM. We found that the DLM using raw ECG signals performed better than MLMs relying solely on ECG features. Although the DLM did not surpass the performance of MLMs that incorporated both patient characteristics and ECG features, the utility of these models will be in identifying possible new cases of HT rather than flagging patients with known thyroid disease. We thus focused on the DLM using raw ECG signals as our primary model.

**Fig. 3:**
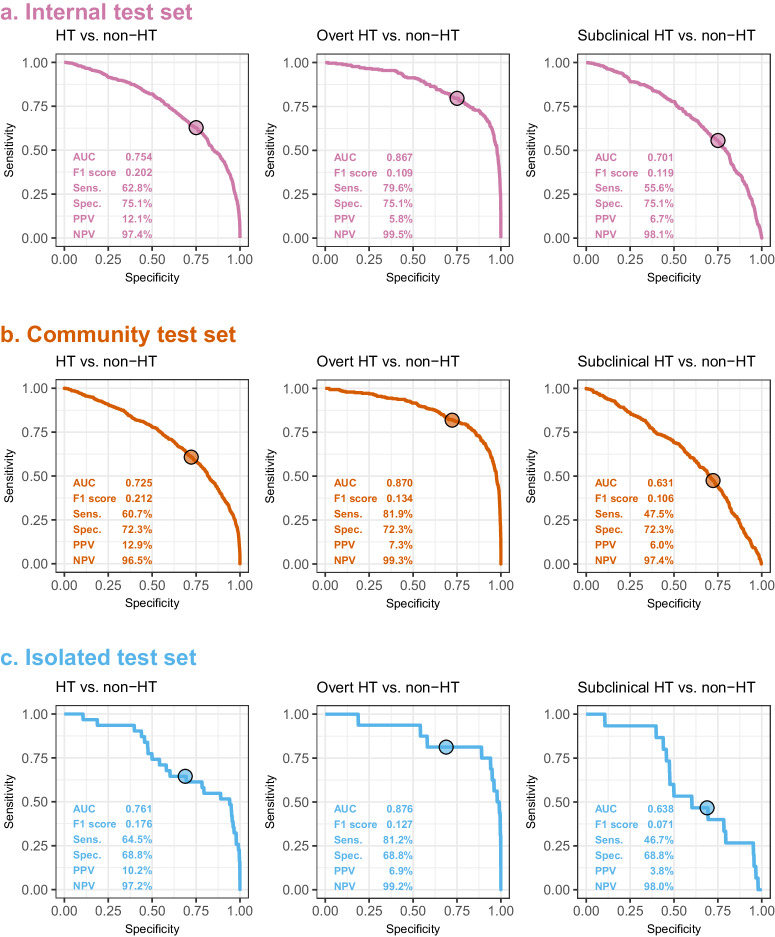
The ROC curve of DLM predictions based on ECG to detect hyperthyroidism (HT), overt HT, and subclinical HT. Overt HT was defined as a free T4 of ≥1.78 ng/dL, and the ECGs without corresponding free T4 tests were excluded. The operating point was selected based on the maximum of Yunden’s index in the validation set for detecting HT compared to non-HT and presented with a circle. Since all analyses shared the same operating point, each test set’s specificities were equal using the same control group (non-HT). The area under ROC curve (AUC), F1 score, sensitivity (Sens.), specificity (Spec.), positive predictive value (PPV), and negative predictive value (NPV) were calculated based on it. Reddish-purple, vermillion, and sky blue represent the results for the internal test set (**a**), community test set (**b**), and isolated test set (**c**), respectively.

As shown in Fig. 3 for the combined detection of overt and subclinical HT, AI-ECG achieved AUCs of 0.754/0.725/0.761 in the internal/community/isolated test sets. To strike a balance between sensitivity and specificity, we defined a single threshold that maximized the Youden index in the validation set of Hospital A as the positive cutoff point. Consequently, we obtained sensitivities of 62.8%, 60.7%, and 64.5%, and specificities of 75.1%, 72.3%, and 68.8% in the internal, community, and isolated test sets. When we applied the same threshold to only overt HT, the sensitivities were improved to ≥79.6% with better AUC (0.867–0.876). The stratified analyses for severe and mild HT are presented in Supplementary Fig. 2. As expected, the performance of the DLM was better in patients with more severe HT.

**Fig. 4:**
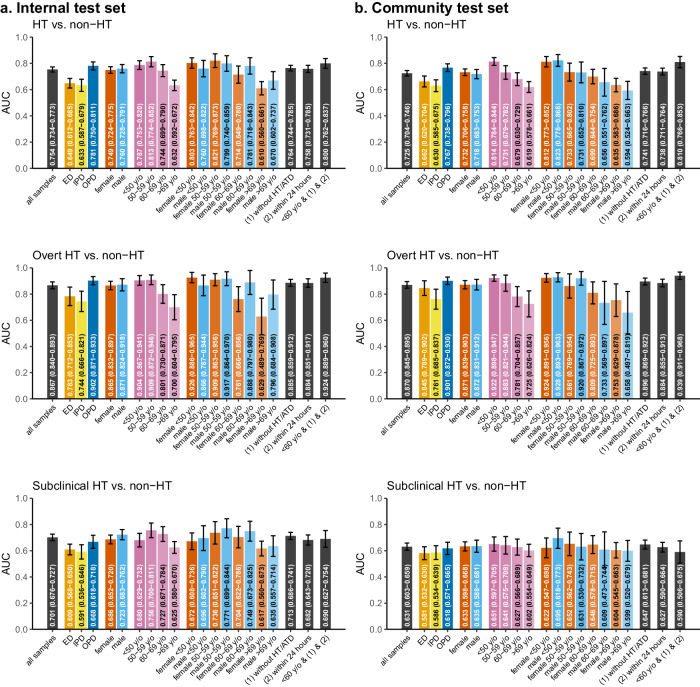
Stratified analysis of selected patient characteristics for AI-ECG performance in predicting hyperthyroidism (HT), overt HT, and subclinical HT. The performance is presented in bar charts and error bar, which represent the area under ROC curve (AUC) and 95% confidence intervals (CI). The analyses were stratified by data source (orange, yellow, and blue for emergency department [ED], inpatient department [IPD], and outpatient department [OPD]), sex (vermillion and sky blue for female and male), age (shades of reddish-purple from dark to light representing younger to older age), and HT information (1 and 2 representing without HT/ATD history and ECG and TSH within 24 h, in black). The black bars on the right side represent those meeting <60 y/o and conditions 1 and 2. The isolated test set was excluded from this analysis due to its small sample size. We presented the performance in internal test set (**a**) and, community test set (**b**), respectively.

As shown in Fig. 4 and Supplementary Fig. 3, subgroups with particularly high AUCs included patients younger than 60 years old, exclusion of patients with a prior history of HT/ATD, and ECG-TSH pairs more than one day apart. Stratified analyses for disease histories demonstrated that AI-ECG performed better in patients without multiple underlying diseases (Supplementary Fig. 4). The improved performance of AI-ECG in detecting HT among outpatients and patients without a history of cardiovascular diseases underscored the need to select the target population carefully for future applications of AI-ECG.

### HT-related AI-ECG features and associated outcomes

**Fig. 5:**
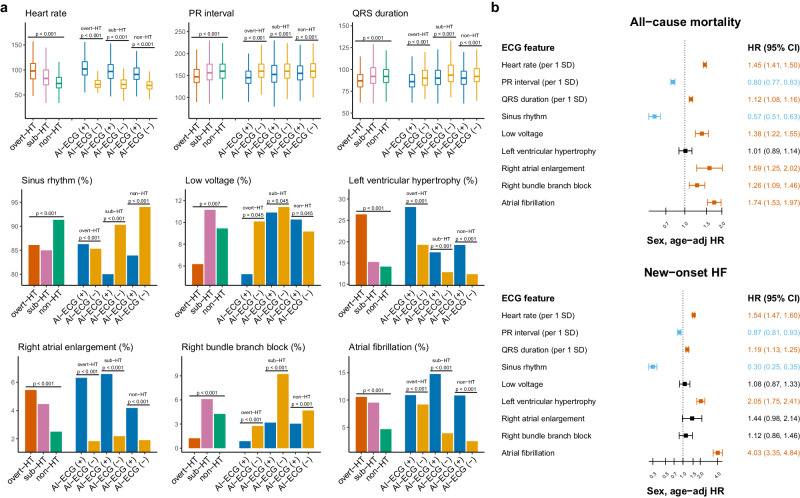
Significant hyperthyroidism (HT) related ECG morphology analysis on adverse outcomes. **a** Distribution of ECG morphologies in overt HT, subclinical HT, and non-HT groups stratified by AI-ECG. This analysis presents the differences in ECG morphologies among different groups, with each group further divided into AI-ECG(+) [representing predicted probabilities greater than the operational cutoff] and AI-ECG(−) [representing predicted probabilities less than the operational cutoff]. For continuous variables, we use boxplots to illustrate their distributions, adjusting for hospitals using linear regression. For categorical variables, we use barplots to depict proportions, adjusting for hospitals using logistic regression. Vermillion, reddish-purple, and bluish-green describe the overt HT, subclinical HT, and non-HT groups, respectively. Blue and orange represent AI-ECG(+) and AI-ECG(−). **b** Risk analysis of selected ECG morphologies on adverse outcomes. This analysis was conducted using the Cox proportional hazard model and combines results from all hospitals. Hazard ratios were adjusted for hospital, sex, and age. The square and error bar represent the hazard ratios and corresponding 95% confidence intervals (CI). Vermillion, black, and sky blue bars denote significantly positive, non-significant, and negative associations, respectively, with the corresponding outcomes. In this analysis, the standard deviations (SD) of heart rate, PR interval, and QRS duration were 19.5, 31.8, and 17.4, respectively.

Supplementary Table 5 shows the ECG characteristics in patients with overt HT, subclinical HT, and non-HT, and Supplementary Table 6 shows the ECG characteristics stratified by AI-ECG. The ECG features significantly associated with laboratory-based thyroid function tests and AI-ECG predictions are summarized in Fig. 5A. ECGs in HT were less likely to exhibit sinus rhythm, low voltage, shorter PR interval, and QRS duration and more likely to exhibit rapid heart rate, left ventricular hypertrophy, right atrial enlargement, and atrial fibrillation, especially in overt HT. Patients with AI-ECG-based HT (positive AI-ECG) in each subgroup were more like overt HT, and patients with AI-ECG-based non-HT (negative AI-ECG) were more like non-HT. Supplementary Table 7 compares each AI-ECG group’s ECG features based on thyroid function. For instance, the mean heart rate in the positive AI-ECG subgroup ranged from 94.6 to 105.1, significantly higher than the negative AI-ECG subgroup (70.2-75.4). This shows that AI-ECG is attuned to specific changes seen in HT-affected hearts.

Figure 5B shows the association between these HT-related ECG characteristics and adverse outcomes. These AI-ECG-identified HT-related ECG changes, including faster heart rate, shorter PR interval, less sinus rhythm, higher voltage, more left ventricular hypertrophy, right atrial enlargement, and atrial fibrillation, were significant risk predictors for adverse outcomes. However, two HT-related ECG changes--shorter QRS duration and less right bundle branch block--were associated with better outcomes. An integration analysis might be necessary to explore the relationship between AI-ECG-based HT and these outcomes.

### Outcome analysis of HT and AI-ECG

**Fig. 6:**
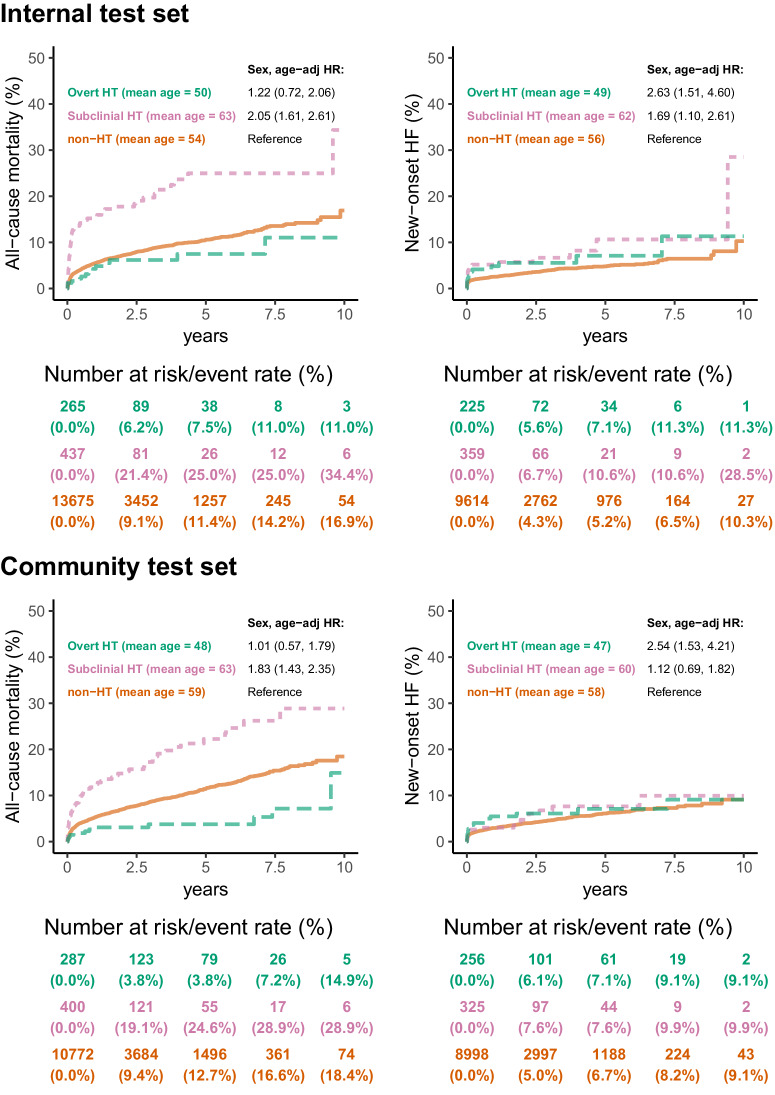
The Kaplan–Meier curve analysis was stratified by laboratory-based thyroid function tests on all-cause mortality and new-onset heart failure (HF). Patients with a prior history of HF were excluded for the analysis of new-onset HF. Vermillion dashed line, reddish-purple dotted line, and bluish-green solid line represent the overt HT, subclinical HT, and non-HT groups, respectively. We have also highlighted the mean age for each group, as the overt HT group is relatively younger than the other groups. This age difference results in a notably higher sex and age-adjusted hazard ratio (HR), especially for new-onset HF. The table displays the at-risk population and cumulative risk for the specified time intervals, categorized by AI-ECG positive and negative.

**Fig. 7:**
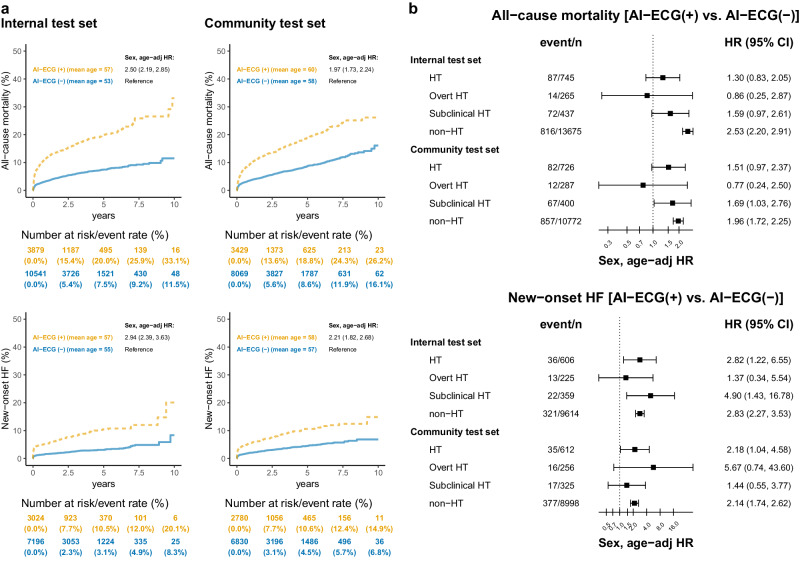
Long-term incidence of developing all-cause mortality and new-onset heart failure (HF) stratified by AI-ECG prediction. **a** The Kaplan–Meier curve analysis stratified by AI-ECG prediction. For the new-onset HF analysis, we excluded patients with a prior history of HF. Yellow dashed line and blue solid line represent AI-ECG (+) [indicating a predicted probability greater than the operational cutoff] and AI-ECG (−) [indicating a predicted probability less than the operational cutoff], respectively. The hazard ratio (HR) presented here has been adjusted for sex and age using a Cox proportional hazards model. The table displays the at-risk population and cumulative risk for the specified time intervals in AI-ECG positive and negative patients. **b** Forest plot illustrating the risk of AI-ECG (+) compared to AI-ECG (−) stratified by hyperthyroidism (HT) and non-HT. The HT group includes overt and subclinical HT (Note: Some cases do not belong to either group due to a lack of free T4 results). We provide the event count and total population for each subgroup. The HR presented here was also adjusted for sex and age using a Cox proportional hazards model. In the figure, the black square represents the point estimate of the HR, while the error bars indicate the 95% confidence intervals (CI).

The average baseline age of the overt, subclinical, and non-HT groups were 49–50, 62–63, and 54–56, with a median follow-up of 1.15 years (interquartile range: 0.06–3.52 years). After adjusting for age and gender, a significantly higher risk of all-cause mortality (HR: 2.05, 95% CI: 1.61–2.61) in the subclinical HT group and a much higher risk of new-onset HF (HR: 2.63, 95% CI: 1.51–4.60) in the overt HT group were found, as shown in Fig. 6. These findings were also validated in the independent community test set with a median follow-up of 2.11 years (interquartile range: 0.50–4.50 years). Figure 7A shows the associations between AI-ECG stratification and adverse outcomes. With similar baseline ages, patients with positive AI-ECGs for HT carried a significantly higher risk of all-cause mortality (HR: 1.97–2.50) and new-onset HF (HR: 2.21–2.94). We also stratified these HT and non-HT patients to assess Al-ECG’s ability to identify previvors of HF and mortality. As shown in Fig. 7B, subclinical HT patients with positive AI-ECG had a significantly higher risk of all-cause mortality and new-onset HF despite the small sample size. Of interest, non-HT patients with positive AI-ECG for HT also demonstrated consistently higher all-cause mortality and new-onset HF.

## Discussion

In this study, we developed an AI-ECG and assessed its diagnostic accuracy and prediction of previvors of adverse outcomes in HT. We utilized a DLM to integrate HT-related ECG features to detect patients with structural or functional cardiac changes. More than 25,000 patients with paired TSH tests and ECG from three independent hospitals were used to evaluate the diagnostic accuracy of AI-ECG for HT detection. AUCs of 0.867–0.876 was achieved for overt HT. AI-ECG also added value by predicting all-cause mortality and new-onset HF independent of thyroid function. To our knowledge, this is the first AI-ECG study to conduct outcome analysis for HT.

For diagnostic accuracy, this study achieved comparable performance in detecting overt HT compared to another recently published study^10^. The AUC of >0.86 in overt HT suggested that AI-ECG may be part of a non-invasive workflow for the early diagnosis of overt HT, especially in patients with age less than 60 years and the absence of multiple co-morbid diseases (much higher AUC of >0.924). Among HT-related AI-ECG features, increased heart rate and shortening of the PR and QRS intervals were associated with overt HT, consistent with previous studies^16–18^. Since sustained HT may lead to atrial fibrillation and thickened heart muscle^19^, these corresponding ECG features were also highlighted. The AI-ECG might use these features to identify ECGs with HT characteristics. However, our AI-ECG system did not identify approximately 20% of overt HT patients. Given that functional and structural heart changes may be related to the cumulative duration of high circulating thyroid hormone exposure^20,21^, these patients may comprise less severity or shorter duration of overt HT. Like most tests, AI-ECG may have less difficulty identifying patients with more severe HT than subclinical HT.

Despite the satisfactory sensitivity and specificity of our AI-ECG for HT, we acknowledge that the low prevalence of HT (~5%), especially overt HT (~2%) in this study, may lead to lower positive predictive values (<15%). The relatively higher false positive rate of AI-ECG for HT may indeed cause both physician’s and patient’s anxiety, inconvenience, confusion, and unnecessary examination and cost for patients. Prior studies of AI-ECG have also encountered similarly high false positives^9^. However, these studies have also consistently found a correlation between false positives and previvors of other cardiovascular diseases, such as AI-ECG based dyskalemia^22^, left ventricular dysfunction^23,24^, and left atrium enlargement^25^. In identifying disease previvors^9^, we also validated that patients with positive AI-ECG had a 1.97–2.94 fold risk for developing all-cause mortality and new-onset HF compared to those with negative AI-ECG, especially in patients with normal thyroid function. Although the sensitivity of ~50% for subclinical HT detection by AI-ECG may seem low initially, those with concerning AI-ECG features like overt HT had a higher risk of all-cause mortality and new-onset HF^26^. Our outcome analysis also demonstrated that those with negative AI-ECGs had a lower risk of future complications in the HT subgroup, demonstrating AI-ECG’s value on top of existing laboratory-based thyroid function tests. The clinical implementation of AI-ECG for HT may also confer risk stratification benefits.

Our rhythm analysis found that AI-ECG identified HT through features such as structural heart disease, rapid heart rate, and atrial fibrillation, similar to left ventricular dysfunction-related features reported in previous studies on AI-ECG^27^. These findings on AI-ECG for HT may account for the significant association between AI-ECG positivity and new-onset HF, independent of thyroid function tests. It is well-known that patients with overt HT are at an increased risk of subsequent HF if left untreated, and the pathophysiological mechanisms are indeed related to structural abnormalities and atrial fibrillation^2^. In particular, atrial fibrillation tends to occur frequently in HT with a prevalence rate of 10–15%, also shown in our study (10% of AF in HT). Recently, an atrial fibrillation-specific AI-ECG algorithm was examined in HT patients to identify HT-related atrial fibrillation^28^. Moreover, up to two-thirds of HT-related atrial fibrillation may converted to sinus rhythm after ATD treatment^29^, which was also validated in this study. Therefore, the correlation between AI-ECG positivity and new-onset HF was reasonable. While the proposed AI-ECG for HT shares similarities with previous AI-ECG models for HF, the distinguishing ECG features between HT and HF, such as a shorter PR interval in HT^6^ and a prolonged PR interval in HF^30^, can be accurately identified by the AI-ECG due to its ability to detect subtle differences. The use of AI-ECG for HT may assist physicians in the differential diagnosis of HF.

The leading clinical utility of this HT AI-ECG system may be opportunistic screening. The concept of opportunistic screening draws inspiration from radiology, where some incidental findings from radiologic imaging conducted for nonspecific reasons can lead to better prognoses through early intervention^31^. Although HT has also been estimated to have an underdiagnosis rate exceeding two-thirds^4^, the US Preventive Services Task Force has recommended against systematic thyroid testing for asymptomatic adults^32^ to prevent harm that may arise from the cascade of care to both patients and clinical providers^33^. Currently, applying an AI model for opportunistic screening has been proposed to address the issue of underdiagnoses in diseases^34,35^ such as osteoporosis^36^. Based on the current study, AI holds tremendous potential as a tool for opportunistic screening of HT. It should be emphasized that the AI-ECG may be more suitable in outpatient departments, given its better performance in those settings. Considering that three million ECGs are conducted worldwide daily^37^, coupled with the high accuracy of HT detection provided by AI-ECG, routine ECG examinations can become a giant net to capture high-risk patients who may benefit from further thyroid testing.

There were some limitations to this study. First, the retrospective design could not answer how many unrecognized HT patients may be detected by passive AI-ECG analysis. A prospective study embedding AI-ECG into a hospital information system should be conducted. Second, it has been shown that the external validation performance of AI-ECG for detecting left ventricular dysfunction (AUC = 0.82 and sensitivity = 27%)^38^ significantly differed from that during model development (AUC = 0.93 and sensitivity = 86%)^39^, which may be attributed to substantial differences in the study populations (prevalence = 0.6% vs. 7.8%). Despite additional external validation conducted in a community hospital and an island hospital in this study, the performance of the DLM may not be generalizable.

In summary, an AI-ECG may become a powerful, non-invasive, bedside tool for potentially detecting HT and predicting adverse cardiovascular outcomes and previvors, especially in patients with ECG abnormalities. For patients with a positive prediction by AI-ECG, a laboratory-based thyroid function test is still warranted for confirmation of HT. A large prospective cohort study to validate such a digital augmentation workflow is warranted.

### Supplementary information

Supplementary Information

Reporting summary

## Data Availability

The authors cannot publicly share the raw ECG signals due to the difficulty of de-identifying them. These signals are securely stored on the servers of Tri-Service General Hospital and may be made available to third parties under a data-sharing agreement for reasonable requests. Access requests should be directed to C. Lin (e-mail: xup6fup@mail.ndmctsgh.edu.tw) or S.H. Lin. Additionally, de-identified tabulated data used for generating statistical charts has been made publicly accessible in the reference (https://github.com/xup6fup/ECG-for-HT)^40^. The numerical data presented in Figs. 2 and 4 can be found in above de-identified tabulated data in github repository, and the exact p-values presented in Fig. 5 can be found in the Supplementary Tables 5 and 7.

## Code availability

The R-based custom code for model training, statistical analysis, and generating Figs. 1–7 is publicly available at the reference (https://github.com/xup6fup/ECG-for-HT)^40^.
