# Supplementary material for: Artificial intelligence-enabled electrocardiography contributes to hyperthyroidism detection and outcome prediction
Source: Commun Med (Lond). 2024 Mar 12;4:42. doi: 10.1038/s43856-024-00472-4 (PMC10933352; doi:10.1038/s43856-024-00472-4)
Supplement: Supplementary file 1 — Supplementary Information [file 43856_2024_472_MOESM1_ESM.pdf]

Artificial intelligence enabled electrocardiography contributes to hyperthyroidism detection and outcome prediction

Supplementary Materials

1. Supplementary tables

Supplementary Table 1 | Corresponding patient characteristics among three datasets in hospital A (academic medical center).

Supplementary Table 2 | Corresponding patient characteristics among hospital A (academic medical center), hospital B (community hospital), and hospital C (local hospital in outlying island).

Supplementary Table 3 | The corresponding patients' characteristics of hyperthyroidism (HT) and non-HT in test sets.

Supplementary Table 4 | Corresponding patient characteristics of overt hyperthyroidism (HT) and subclinical HT.

Supplementary Table 5 | Detailed ECG features in overt hyperthyroidism (HT), subclinical HT, and non-HT cases.

Supplementary Table 6 | Detailed ECG features stratified by AI-ECG.

Supplementary Table 7 | Detailed ECG features stratified by AI-ECG in overt hyperthyroidism (HT), subclinical HT, and non-HT cases.

2. Supplementary figures

Supplementary Figure 1 | The implementation of our deep learning model.

Supplementary Figure 2 | The ROC curve of DLM predictions based on ECG to detect hyperthyroidism (HT), overt HT, and subclinical HT stratified by severe (TSH ≤0.05 μIU/mL) and mild (TSH 0.06-0.50 μIU/mL) condition.

Supplementary Figure 3 | The ROC curve of predictions based on ECG and demography to detect hyperthyroidism (HT), overt HT, and subclinical HT.

Supplementary Figure 4 | Stratified analysis for AI-ECG performance for predicting hyperthyroidism (HT), overt HT, and subclinical HT.

Supplementary Table 1 | Corresponding patient characteristics among three datasets in hospital A (academic medical center).

	Training set (n = 23728)	Validation set (n = 9518)	Internal test set (n = 14420)	p-value
**Data source**				0.702
ED	3817(16.1%)	1584(16.6%)	2332(16.2%)	
IPD	3642(15.3%)	1446(15.2%)	2170(15.0%)	
OPD	16269(68.6%)	6488(68.2%)	9918(68.8%)	
**HT information**				
History of HT	563(2.4%)	238(2.5%)	326(2.3%)	0.486
History of ATD	231(1.0%)	106(1.1%)	112(0.8%)	2.37e-02
Within 1 day	17950(75.6%)	7159(75.2%)	10904(75.6%)	0.691
**Demography**				
Sex (male)	11632(49.0%)	4583(48.2%)	6991(48.5%)	0.300
Age (years)	54.2±18.4	54.2±18.4	54.2±18.4	0.945
BMI (kg/m^2)	24.1±4.1	24.1±4.2	24.2±4.1	0.460
**Disease history**				
DM	3253(13.7%)	1319(13.9%)	2043(14.2%)	0.454
HTN	869(3.7%)	350(3.7%)	529(3.7%)	0.998
HLP	4921(20.7%)	1973(20.7%)	2973(20.6%)	0.957
CKD	2199(9.3%)	889(9.3%)	1361(9.4%)	0.857
AMI	274(1.2%)	121(1.3%)	186(1.3%)	0.442
STK	1895(8.0%)	751(7.9%)	1179(8.2%)	0.695
CAD	2702(11.4%)	1093(11.5%)	1636(11.3%)	0.946
HF	1199(5.1%)	471(4.9%)	705(4.9%)	0.764
Afib	750(3.2%)	311(3.3%)	444(3.1%)	0.716
COPD	2014(8.5%)	795(8.4%)	1203(8.3%)	0.857

Abbreviations: ED, emergency department; IPD, inpatient department; OPD, outpatient department; HT, hyperthyroidism; ATD, anti-thyroid drug; BMI, body mass index; DM, diabetes mellitus; HTN, hypertension; HLP, hyperlipidemia; CKD, chronic kidney disease; AMI, acute myocardial infraction; STK, stroke; CAD, coronary artery disease; HF, heart failure; Afib, atrial fibrillation; COPD, chronic obstructive pulmonary disease.

Supplementary Table 2 | Corresponding patient characteristics among hospital A (academic medical center), hospital B (community hospital), and hospital C (local hospital in outlying island).

	hospital A (n = 47666)	hospital B (n = 11498)	hospital C (n = 596)	p-value
**Data source**				2.77e-110
ED	7733(16.2%)	2098(18.2%)	0(0.0%)	
IPD	7258(15.2%)	2312(20.1%)	0(0.0%)	
OPD	32675(68.5%)	7088(61.6%)	596(100.0%)	
**HT information**				
History of HT	1127(2.4%)	609(5.3%)	21(3.5%)	1.73e-61
History of ATD	449(0.9%)	203(1.8%)		8.23e-15
Within 1 day	36013(75.6%)	7649(66.5%)	507(85.1%)	3.42e-94
**Demography**				
Sex (male)	23206(48.7%)	4999(43.5%)	288(48.3%)	1.29e-22
Age (years)	54.2±18.4	58.5±19.3	59.4±17.7	2.12e-113
BMI (kg/m^2)	24.1±4.1	24.1±4.2	24.4±4.3	0.263
**Disease history**				
DM	6615(13.9%)	2699(23.5%)	130(21.8%)	1.77e-143
HTN	1748(3.7%)	793(6.9%)	2(0.3%)	3.73e-57
HLP	9867(20.7%)	4397(38.2%)	152(25.5%)	2.23e-308
CKD	4449(9.3%)	1677(14.6%)	95(15.9%)	1.71e-64
AMI	581(1.2%)	158(1.4%)	5(0.8%)	0.269
STK	3825(8.0%)	1526(13.3%)	49(8.2%)	3.25e-68
CAD	5431(11.4%)	2265(19.7%)	65(10.9%)	5.2e-124
HF	2375(5.0%)	1036(9.0%)	37(6.2%)	8.6e-61
Afib	1505(3.2%)	683(5.9%)	30(5.0%)	5.94e-45
COPD	4012(8.4%)	2057(17.9%)	56(9.4%)	4.55e-197

Abbreviations: ED, emergency department; IPD, inpatient department; OPD, outpatient department; HT, hyperthyroidism; ATD, anti-thyroid drug; BMI, body mass index; DM, diabetes mellitus; HTN, hypertension; HLP, hyperlipidemia; CKD, chronic kidney disease; AMI, acute myocardial infraction; STK, stroke; CAD, coronary artery disease; HF, heart failure; Afib, atrial fibrillation; COPD, chronic obstructive pulmonary disease.

Supplementary Table 3 | The corresponding patients' characteristics of hyperthyroidism (HT) and non-HT in test sets.

	Internal test set			Community test set			Isolated test set		
	HT (n = 745)	non-HT (n = 13675)	p-value	HT (n = 726)	non-HT (n = 10772)	p-value	HT (n = 31)	non-HT (n = 565)	p-value
**HT severity**									
Severe HT	472(63.4%)			487(67.1%)			20(64.5%)		
Mild HT	273(35.6%)			239(32.9%)			11(35.5%)		
**fT4 stratification**									
Overt HT	265(37.7%)			287(41.8%)			16(51.6%)		
Subclinical HT	437(62.3%)			400(58.2%)			15(48.4%)		
without fT4	43			39			0		
**HT severity and fT4 stratification**									
Severe overt HT	241(34.3%)			261(38.0%)			15(48.4%)		
Severe subclinical HT	206(29.3%)			201(29.3%)			5(16.1%)		
Severe overt HT	24(3.4%)			26(3.8%)			1(3.2%)		
Severe subclinical HT	231(32.9%)			199(29.0%)			10(32.3%)		
without fT4	43			39			0		
**Data source**			4.62e-60			2.99e-16			1.000
ED	259(34.8%)	2073(15.2%)		204(28.1%)	1894(17.6%)		0(0.0%)	0(0.0%)	
IPD	167(22.4%)	2003(14.6%)		175(24.1%)	2137(19.8%)		0(0.0%)	0(0.0%)	
OPD	319(42.8%)	9599(70.2%)		347(47.8%)	6741(62.6%)		31(100.0%)	565(100.0%)	
**HT information**									
History of HT	119(16.0%)	207(1.5%)	2.12e-147	222(30.6%)	387(3.6%)	9.52e-217	10(32.3%)	11(1.9%)	7.50e-09
History of ATD	51(6.8%)	61(0.4%)	1.22e-83	91(12.5%)	112(1.0%)	1.06e-114			
Within 1 day	413(55.4%)	10491(76.7%)	1.25e-39	441(60.7%)	7208(66.9%)	6.49e-04	25(80.6%)	482(85.3%)	0.442
**Demography**									
Sex (male)	266(35.7%)	6725(49.2%)	7.75e-13	258(35.5%)	4741(44.0%)	8.25e-06	12(38.7%)	276(48.8%)	0.271
Age (years)	57.7±18.6	54.0±18.3	1.13e-07	56.9±19.5	58.6±19.2	2.20e-02	51.6±15.4	59.8±17.7	1.22e-02
BMI (kg/m^2)	23.8±4.2	24.2±4.1	1.18e-02	24.0±4.2	24.1±4.2	0.643	24.7±4.3	24.3±4.4	0.647
**Disease history**									
DM	129(17.3%)	1914(14.0%)	1.14e-02	172(23.7%)	2527(23.5%)	0.886			
HTN	45(6.0%)	484(3.5%)	4.06e-04	64(8.8%)	729(6.8%)	3.51e-02			
HLP	125(16.8%)	2848(20.8%)	7.83e-03	254(35.0%)	4143(38.5%)	6.22e-02			
CKD	116(15.6%)	1245(9.1%)	4.13e-09	107(14.7%)	1570(14.6%)	0.904			
AMI	20(2.7%)	166(1.2%)	5.32e-04	18(2.5%)	140(1.3%)	8.22e-03			
STK	70(9.4%)	1109(8.1%)	0.212	81(11.2%)	1445(13.4%)	8.27e-02			
CAD	95(12.8%)	1541(11.3%)	0.214	134(18.5%)	2131(19.8%)	0.385			
HF	54(7.2%)	651(4.8%)	2.17e-03	85(11.7%)	951(8.8%)	8.72e-03			
Afib	46(6.2%)	398(2.9%)	5.11e-07	59(8.1%)	624(5.8%)	1.00e-02			
COPD	70(9.4%)	1133(8.3%)	0.286	119(16.4%)	1938(18.0%)	0.276			

Abbreviations: ED, emergency department; IPD, inpatient department; OPD, outpatient department; ATD, anti-thyroid drug; BMI, body mass index; DM, diabetes mellitus; HTN, hypertension; HLP, hyperlipidemia; CKD, chronic kidney disease; AMI, acute myocardial infraction; STK, stroke; CAD, coronary artery disease; HF, heart failure; Afib, atrial fibrillation; COPD, chronic obstructive pulmonary disease.

Supplementary Table 4 | Corresponding patient characteristics of overt hyperthyroidism (HT) and subclinical HT.

	Internal test set			Community test set			Isolated test set		
	Overt HT (n = 265)	Subclinical HT (n = 437)	p-value	Overt HT (n = 287)	Subclinical HT (n = 400)	p-value	Overt HT (n = 16)	Subclinical HT (n = 15)	p-value
**Data source**			2.38e-17			1.33e-10			1.000
ED	60(22.6%)	195(44.6%)		64(22.3%)	136(34.0%)		0(0.0%)	0(0.0%)	
IPD	44(16.6%)	122(27.9%)		47(16.4%)	122(30.5%)		0(0.0%)	0(0.0%)	
OPD	161(60.8%)	120(27.5%)		176(61.3%)	142(35.5%)		16(100.0%)	15(100.0%)	
**HT information**									
History of HT	48(18.1%)	67(15.3%)	0.334	92(32.1%)	127(31.8%)	0.932	4(25.0%)	6(40.0%)	0.458
History of ATD	31(11.7%)	18(4.1%)	1.33e-04	49(17.1%)	42(10.5%)	1.22e-02			
Within 1 day	170(64.2%)	204(46.7%)	6.89e-06	185(64.5%)	229(57.2%)	5.68e-02	13(81.2%)	12(80.0%)	1.000
**Demography**									
Sex (male)	82(30.9%)	171(39.1%)	2.85e-02	95(33.1%)	153(38.2%)	0.166	4(25.0%)	8(53.3%)	0.106
Age (years)	50.1±17.2	62.6±17.7	5.78e-19	47.9±17.7	62.7±18.0	6.85e-25	43.5±12.8	60.2±13.4	1.47e-03
BMI (kg/m^2)	23.2±4.1	24.0±4.2	1.14e-02	23.9±4.2	24.2±4.2	0.391	23.9±4.5	25.5±4.1	0.151
**Disease history**									
DM	28(10.6%)	97(22.2%)	9.43e-05	40(13.9%)	126(31.5%)	1.14e-07	2(12.5%)	6(40.0%)	0.113
HTN	18(6.8%)	26(5.9%)	0.655	21(7.3%)	40(10.0%)	0.223	0(0.0%)	0(0.0%)	1.000
HLP	32(12.1%)	86(19.7%)	9.01e-03	71(24.7%)	175(43.8%)	2.96e-07	1(6.2%)	7(46.7%)	1.55e-02
CKD	17(6.4%)	99(22.7%)	1.95e-08	16(5.6%)	89(22.2%)	2.09e-09	1(6.2%)	3(20.0%)	0.333
AMI	1(0.4%)	19(4.3%)	2.17e-03	2(0.7%)	16(4.0%)	7.51e-03	0(0.0%)	0(0.0%)	1.000
STK	15(5.7%)	53(12.1%)	4.98e-03	9(3.1%)	69(17.2%)	8.87e-09	0(0.0%)	2(13.3%)	0.226
CAD	23(8.7%)	68(15.6%)	8.51e-03	27(9.4%)	99(24.8%)	2.98e-07	0(0.0%)	4(26.7%)	4.34e-02
HF	15(5.7%)	38(8.7%)	0.140	23(8.0%)	58(14.5%)	9.33e-03	0(0.0%)	0(0.0%)	1.000
Afib	16(6.0%)	28(6.4%)	0.845	30(10.5%)	28(7.0%)	0.108	1(6.2%)	1(6.7%)	1.000
COPD	16(6.0%)	48(11.0%)	2.73e-02	22(7.7%)	87(21.8%)	6.25e-07	1(6.2%)	1(6.7%)	1.000

Abbreviations: ED, emergency department; IPD, inpatient department; OPD, outpatient department; HT, hyperthyroidism; ATD, anti-thyroid drug; BMI, body mass index; DM, diabetes mellitus; HTN, hypertension; HLP, hyperlipidemia; CKD, chronic kidney disease; AMI, acute myocardial infraction; STK, stroke; CAD, coronary artery disease; HF, heart failure; Afib, atrial fibrillation; COPD, chronic obstructive pulmonary disease.

Supplementary Table 5 | Detailed ECG features in overt hyperthyroidism, subclinical HT, and non-HT cases.

	overt HT (n = 568)	subclinical HT (n = 852)	non-HT (n = 25012)	p-value	Post hoc test
**ECG measurement**					
Heart rate	99.4±25.4	87.5±25.2	76.7±18.8	6.68e-219	abc
PR interval	149.3±27.1	159.0±35.5	161.4±31.8	1.05e-18	ab
QRS duration	89.1±15.7	96.5±20.8	94.9±17.3	6.23e-16	abc
QT interval	353.5±49.3	383.6±57.1	394.0±43.5	8.05e-110	abc
QTc interval	444.5±37.4	451.7±46.9	438.0±38.7	4.77e-26	c
P waves axes	52.1±30.3	49.6±38.6	50.0±31.1	0.271	ab
QRS waves axes	51.1±35.0	39.7±48.6	43.1±43.8	5.57e-06	ab
T waves axes	41.6±42.7	50.9±62.3	41.3±43.0	3.27e-09	ac
**ECG pattern**					
SR	489(86.1%)	724(85.0%)	22844(91.3%)	2.67e-13	bc
SA	3(0.5%)	1(0.1%)	216(0.9%)	2.67e-02	
SP	2(0.4%)	4(0.5%)	32(0.1%)	2.33e-02	
EAR	7(1.2%)	18(2.1%)	299(1.2%)	5.71e-02	
JER	2(0.4%)	6(0.7%)	91(0.4%)	0.247	
PMR	1(0.2%)	6(0.7%)	115(0.5%)	0.383	
ET	47(8.3%)	81(9.5%)	2078(8.3%)	0.460	
STE	137(24.1%)	180(21.1%)	6551(26.2%)	2.43e-03	c
STD	10(1.8%)	35(4.1%)	492(2.0%)	6.81e-05	ac
ATW	54(9.5%)	103(12.1%)	2834(11.3%)	0.307	
AQW	9(1.6%)	20(2.3%)	313(1.3%)	1.71e-02	c
RSRW	30(5.3%)	40(4.7%)	1005(4.0%)	0.206	
LVOL	35(6.2%)	95(11.2%)	2363(9.4%)	6.53e-03	ab
LAD	13(2.3%)	38(4.5%)	1077(4.3%)	6.05e-02	
RAD	26(4.6%)	23(2.7%)	875(3.5%)	0.168	
LVH	150(26.4%)	130(15.3%)	3544(14.2%)	2.01e-15	ab
RVH	3(0.5%)	5(0.6%)	346(1.4%)	3.26e-02	
LAE	57(10.0%)	98(11.5%)	1994(8.0%)	2.53e-04	c
RAE	31(5.5%)	38(4.5%)	626(2.5%)	2.42e-07	bc
LAA	9(1.6%)	10(1.2%)	159(0.6%)	6.48e-03	b
RAA	13(2.3%)	4(0.5%)	71(0.3%)	7.21e-08	ab
NIVCD	15(2.6%)	41(4.8%)	959(3.8%)	0.111	
LFB	8(1.4%)	28(3.3%)	861(3.4%)	2.96e-02	b
RBBB	7(1.2%)	52(6.1%)	1064(4.3%)	4.75e-05	abc
LBBB	1(0.2%)	13(1.5%)	202(0.8%)	1.84e-02	a
1AVB	13(2.3%)	47(5.5%)	1055(4.2%)	1.24e-02	a
2AVB	1(0.2%)	7(0.8%)	91(0.4%)	8.18e-02	
CAVB	0(0.0%)	0(0.0%)	23(0.1%)	1.000	
AFIB	60(10.6%)	81(9.5%)	1160(4.6%)	2.31e-18	bc
AFLT	3(0.5%)	14(1.6%)	200(0.8%)	3.37e-02	c
SVT	3(0.5%)	3(0.4%)	19(0.1%)	1.36e-03	b
VPE	1(0.2%)	1(0.1%)	6(0.0%)	6.51e-02	
RVPC	2(0.4%)	0(0.0%)	45(0.2%)	0.205	
APC	9(1.6%)	36(4.2%)	591(2.4%)	9.88e-04	ac
VPC	36(6.3%)	52(6.1%)	1213(4.8%)	7.23e-02	

Result of post hoc test: [a]significant difference between overt HT and subclinical HT; [b]significant difference between overt HT and non-HTT; [c]significant difference between subclinical HT and non-HT. Abbreviations: SR, sinus rhythm; SA, sinus arrhythmia; SP, sinus pause; EAR, ectopic atrial rhythm; JER, junctional rhythm; PMR, pacemaker rhythm; ET, early precordial R/S transition; STE, ST elevation; STD, ST depression; ATW, abnormal T wave; AQW, abnormal Q wave; RSRW, RSR' wave; LVOL, low voltage; LAD, left axis deviation; RAD, right axis deviation; LVH, left ventricular hypertrophy; RVH, right ventricular hypertrophy; LAE, left atrial enlargement; RAE, right atrial enlargement; LAA, left atrium abnormality; RAA, right atrium abnormality; NIVCD, nonspecific intraventricular conduction delay; LFB, left fascicular block; RBBB, right bundle branch block; LBBB, left bundle branch block; 1AVB, first degree AV block; 2AVB, second degree AV block; CAVB, complete degree AV block; AFIB, atrial fibrillation; AFLT, atrial flutter; SVT, supraventricular tachycardia; VPE, WPW syndrome; RVPC, ventricular tachycardia; APC, atrial premature complex; VPC, ventricular premature complex.

Supplementary Table 6 | Detailed ECG features stratified by AI-ECG.

	AI-ECG (+)<br>(n = 7504)	AI-ECG (-)<br>(n = 19010)	p-value
**ECG measurement**			
Heart rate	95.5±21.2	70.4±13.2	2.23e-308
PR interval	155.6±30.8	163.1±32.1	7.38e-67
QRS duration	92.9±17.1	95.6±17.5	4.62e-32
QT interval	364.2±44.1	404.1±39.4	2.23e-308
QTc interval	452.0±39.3	433.2±37.6	2.06e-280
P waves axes	51.8±34.3	49.3±30.1	2.88e-09
QRS waves axes	43.5±44.4	43.0±43.5	0.464
T waves axes	45.1±56.5	40.3±37.4	6.66e-16
**ECG pattern**			
SR	6289(83.8%)	17844(93.9%)	7.95e-147
SA	55(0.7%)	166(0.9%)	0.258
SP	14(0.2%)	24(0.1%)	0.242
EAR	130(1.7%)	194(1.0%)	2.01e-06
JER	34(0.5%)	65(0.3%)	0.181
PMR	27(0.4%)	95(0.5%)	0.129
ET	663(8.8%)	1549(8.1%)	6.84e-02
STE	1681(22.4%)	5203(27.4%)	9.40e-17
STD	279(3.7%)	261(1.4%)	4.09e-34
ATW	1008(13.4%)	1992(10.5%)	7.9e-12
AQW	147(2.0%)	196(1.0%)	1.71e-09
RSRW	282(3.8%)	796(4.2%)	0.111
LVOL	750(10.0%)	1746(9.2%)	4.19e-02
LAD	280(3.7%)	852(4.5%)	6.47e-03
RAD	292(3.9%)	634(3.3%)	2.63e-02
LVH	1476(19.7%)	2361(12.4%)	1.29e-51
RVH	121(1.6%)	234(1.2%)	1.49e-02
LAE	908(12.1%)	1250(6.6%)	1.08e-49
RAE	334(4.5%)	362(1.9%)	1.54e-31
LAA	82(1.1%)	96(0.5%)	1.3e-07
RAA	52(0.7%)	36(0.2%)	1.34e-10
NIVCD	271(3.6%)	745(3.9%)	0.240
LFB	236(3.1%)	661(3.5%)	0.178
RBBB	218(2.9%)	907(4.8%)	1.12e-11
LBBB	64(0.9%)	152(0.8%)	0.664
1AVB	286(3.8%)	830(4.4%)	4.27e-02
2AVB	43(0.6%)	56(0.3%)	8.12e-04
CAVB	6(0.1%)	17(0.1%)	0.813
AFIB	830(11.1%)	476(2.5%)	5.99e-185
AFLT	97(1.3%)	120(0.6%)	7.26e-08
SVT	17(0.2%)	8(0.0%)	1.04e-05
VPE	4(0.1%)	4(0.0%)	0.234
RVPC	21(0.3%)	26(0.1%)	1.26e-02
APC	255(3.4%)	386(2.0%)	6.52e-11
VPC	591(7.9%)	714(3.8%)	2.4e-44

According to the predicted probability by AI-ECG, we defined those higher than cut-off point according to Figure 2 as AI-ECG (+), and those lower than cut-off point as AI-ECG (-), respectively. Abbreviations: SR, sinus rhythm; SA, sinus arrhythmia; SP, sinus pause; EAR, ectopic atrial rhythm; JER, junctional rhythm; PMR, pacemaker rhythm; ET, early precordial R/S transition; STE, ST elevation; STD, ST depression; ATW, abnormal T wave; AQW, abnormal Q wave; RSRW, RSR' wave; LVOL, low voltage; LAD, left axis deviation; RAD, right axis deviation; LVH, left ventricular hypertrophy; RVH, right ventricular hypertrophy; LAE, left atrial enlargement; RAE, right atrial enlargement; LAA, left atrium abnormality; RAA, right atrium abnormality; NIVCD, nonspecific intraventricular conduction delay; LFB, left fascicular block; RBBB, right bundle branch block; LBBB, left bundle branch block; 1AVB, first degree AV block; 2AVB, second degree AV block; CAVB, complete degree AV block; AFIB, atrial fibrillation; AFLT, atrial flutter; SVT, supraventricular tachycardia; VPE, WPW syndrome; RVPC, ventricular tachycardia; APC, atrial premature complex; VPC, ventricular premature complex.

Supplementary Table 7 | Detailed ECG features stratified by AI-ECG in overt hyperthyroidism (HT), subclinical HT, and non-HT cases.

	AI-ECG (+) (n = 7474)				AI-ECG (-) (n = 18958)			
	overt HT (n = 459)	subclinical HT (n = 440)	non-HT (n = 6575)	p-value	overt HT (n = 109)	subclinical HT (n = 412)	non-HT (n = 18437)	p-value
**ECG measurement**								
Heart rate	105.1±22.4	99.9±24.9	94.6±20.6	5.76e-28	75.4±22.8	74.3±17.7	70.2±13.0	1.94e-12
PR interval	146.2±25.4	155.1±33.9	156.4±30.9	5.28e-11	162.6±30.1	163.2±36.7	163.1±32.0	0.983
QRS duration	88.0±14.0	93.5±19.4	93.2±17.1	1.53e-09	93.9±20.7	99.7±21.8	95.6±17.4	6.91e-06
QT interval	341.9±41.6	359.1±48.8	366.1±43.5	3.87e-30	401.9±50.0	409.7±53.8	404.0±38.9	1.23e-02
QTc interval	445.2±35.4	453.7±43.0	452.4±39.3	4.41e-04	441.8±44.8	449.6±50.7	432.8±37.1	1.70e-19
P waves axes	53.8±30.1	51.8±41.4	51.6±34.1	0.429	44.7±30.6	47.3±35.2	49.4±30.0	0.112
QRS waves axes	54.2±33.3	40.4±46.5	42.9±44.8	3.09e-07	38.3±39.3	39.0±50.9	43.2±43.4	8.23e-02
T waves axes	39.8±40.7	58.5±71.0	44.5±56.3	4.15e-07	49.1±49.5	42.7±50.4	40.2±37.0	1.92e-02
**ECG pattern**								
SR	396(86.3%)	352(80.0%)	5516(83.9%)	3.34e-02	93(85.3%)	372(90.3%)	17328(94.0%)	8.4e-06
SA	2(0.4%)	1(0.2%)	51(0.8%)	0.429	1(0.9%)	0(0.0%)	165(0.9%)	8.66e-02
SP	2(0.4%)	2(0.5%)	10(0.2%)	0.116	0(0.0%)	2(0.5%)	22(0.1%)	0.140
EAR	6(1.3%)	13(3.0%)	111(1.7%)	0.111	1(0.9%)	5(1.2%)	188(1.0%)	0.795
JER	0(0.0%)	2(0.5%)	32(0.5%)	0.426	2(1.8%)	4(1.0%)	59(0.3%)	9.27e-03
PMR	0(0.0%)	1(0.2%)	26(0.4%)	0.589	1(0.9%)	5(1.2%)	89(0.5%)	6.90e-02
ET	37(8.1%)	41(9.3%)	582(8.9%)	0.790	10(9.2%)	40(9.7%)	1496(8.1%)	0.468
STE	118(25.7%)	90(20.5%)	1466(22.3%)	0.143	19(17.4%)	90(21.8%)	5085(27.6%)	2.31e-03
STD	10(2.2%)	22(5.0%)	246(3.7%)	7.94e-02	0(0.0%)	13(3.2%)	246(1.3%)	9.16e-03
ATW	39(8.5%)	60(13.6%)	905(13.8%)	5.93e-03	15(13.8%)	43(10.4%)	1929(10.5%)	0.533
AQW	8(1.7%)	10(2.3%)	128(1.9%)	0.843	1(0.9%)	10(2.4%)	185(1.0%)	2.51e-02
RSRW	27(5.9%)	23(5.2%)	231(3.5%)	8.93e-03	3(2.8%)	17(4.1%)	774(4.2%)	0.859
LVOL	24(5.2%)	48(10.9%)	675(10.3%)	1.89e-03	11(10.1%)	47(11.4%)	1688(9.2%)	0.280
LAD	6(1.3%)	16(3.6%)	258(3.9%)	1.69e-02	7(6.4%)	22(5.3%)	819(4.4%)	0.338
RAD	22(4.8%)	12(2.7%)	256(3.9%)	0.273	4(3.7%)	11(2.7%)	619(3.4%)	0.734
LVH	129(28.1%)	77(17.5%)	1263(19.2%)	1.08e-05	21(19.3%)	53(12.9%)	2281(12.4%)	9.03e-02
RVH	3(0.7%)	2(0.5%)	116(1.8%)	2.60e-02	0(0.0%)	3(0.7%)	230(1.2%)	0.486
LAE	53(11.5%)	61(13.9%)	790(12.0%)	0.481	4(3.7%)	37(9.0%)	1204(6.5%)	6.58e-02
RAE	29(6.3%)	29(6.6%)	275(4.2%)	8.21e-03	2(1.8%)	9(2.2%)	351(1.9%)	0.813
LAA	9(2.0%)	7(1.6%)	66(1.0%)	8.25e-02	0(0.0%)	3(0.7%)	93(0.5%)	0.695
RAA	12(2.6%)	4(0.9%)	36(0.5%)	6.91e-05	1(0.9%)	0(0.0%)	35(0.2%)	0.223
NIVCD	10(2.2%)	19(4.3%)	242(3.7%)	0.182	5(4.6%)	22(5.3%)	717(3.9%)	0.254
LFB	7(1.5%)	12(2.7%)	217(3.3%)	9.51e-02	1(0.9%)	16(3.9%)	644(3.5%)	0.332
RBBB	4(0.9%)	14(3.2%)	200(3.0%)	2.66e-02	3(2.8%)	38(9.2%)	864(4.7%)	6.66e-05
LBBB	0(0.0%)	5(1.1%)	59(0.9%)	5.81e-02	1(0.9%)	8(1.9%)	143(0.8%)	3.03e-02
1AVB	6(1.3%)	26(5.9%)	254(3.9%)	1.41e-03	7(6.4%)	21(5.1%)	801(4.3%)	0.355
2AVB	1(0.2%)	3(0.7%)	39(0.6%)	0.626	0(0.0%)	4(1.0%)	52(0.3%)	7.33e-02
CAVB	0(0.0%)	0(0.0%)	6(0.1%)	1.000	0(0.0%)	0(0.0%)	17(0.1%)	1.000
AFIB	50(10.9%)	65(14.8%)	711(10.8%)	3.71e-02	10(9.2%)	16(3.9%)	449(2.4%)	2.05e-04
AFLT	3(0.7%)	5(1.1%)	89(1.4%)	0.420	0(0.0%)	9(2.2%)	111(0.6%)	3.46e-03
SVT	2(0.4%)	2(0.5%)	13(0.2%)	0.188	1(0.9%)	1(0.2%)	6(0.0%)	7.74e-03
VPE	1(0.2%)	0(0.0%)	3(0.0%)	0.401	0(0.0%)	1(0.2%)	3(0.0%)	0.105
RVPC	2(0.4%)	0(0.0%)	19(0.3%)	0.499	0(0.0%)	0(0.0%)	26(0.1%)	1.000
APC	8(1.7%)	24(5.5%)	221(3.4%)	8.40e-03	1(0.9%)	12(2.9%)	370(2.0%)	0.333
VPC	30(6.5%)	28(6.4%)	530(8.1%)	0.242	6(5.5%)	24(5.8%)	683(3.7%)	4.82e-02

Abbreviations: SR, sinus rhythm; SA, sinus arrhythmia; SP, sinus pause; EAR, ectopic atrial rhythm; JER, junctional rhythm; PMR, pacemaker rhythm; ET, early precordial R/S transition; STE, ST elevation; STD, ST depression; ATW, abnormal T wave; AQW, abnormal Q wave; RSRW, RSR' wave; LVOL, low voltage; LAD, left axis deviation; RAD, right axis deviation; LVH, left ventricular hypertrophy; RVH, right ventricular hypertrophy; LAE, left atrial enlargement; RAE, right atrial enlargement; LAA, left atrium abnormality; RAA, right atrium abnormality; NIVCD, nonspecific intraventricular conduction delay; LFB, left fascicular block; RBBB, right bundle branch block; LBBB, left bundle branch block; 1AVB, first degree AV block; 2AVB, second degree AV block; CAVB, complete degree AV block; AFIB, atrial fibrillation; AFLT, atrial flutter; SVT, supraventricular tachycardia; VPE, WPW syndrome; RVPC, ventricular tachycardia; APC, atrial premature complex; VPC, ventricular premature complex.

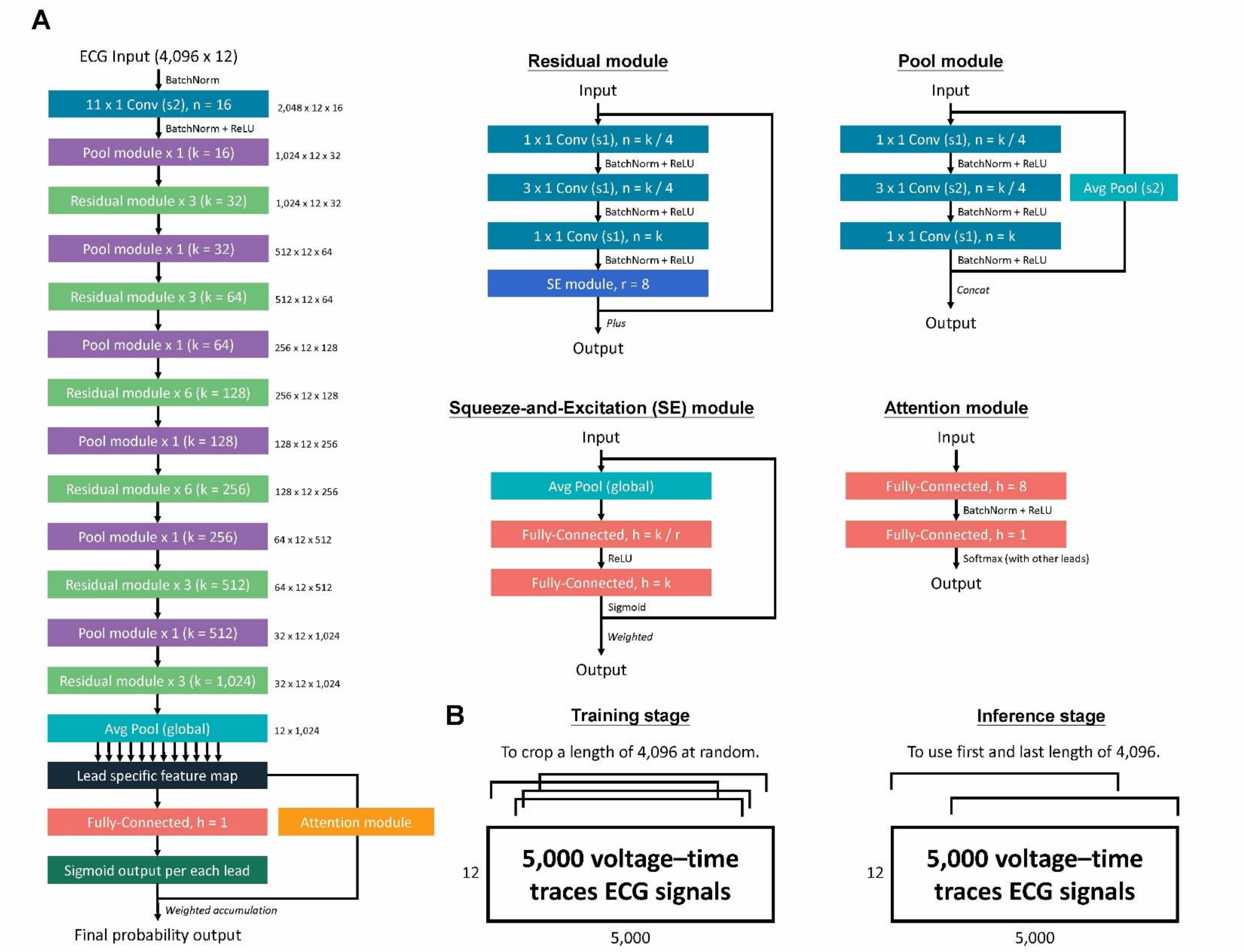

Supplementary Figure 1 | The implementation of our deep learning model. A) The model architectures of deep learning model for analyzing ECG. B) The sampling strategy of ECG in training and inference stage.

Internal test set

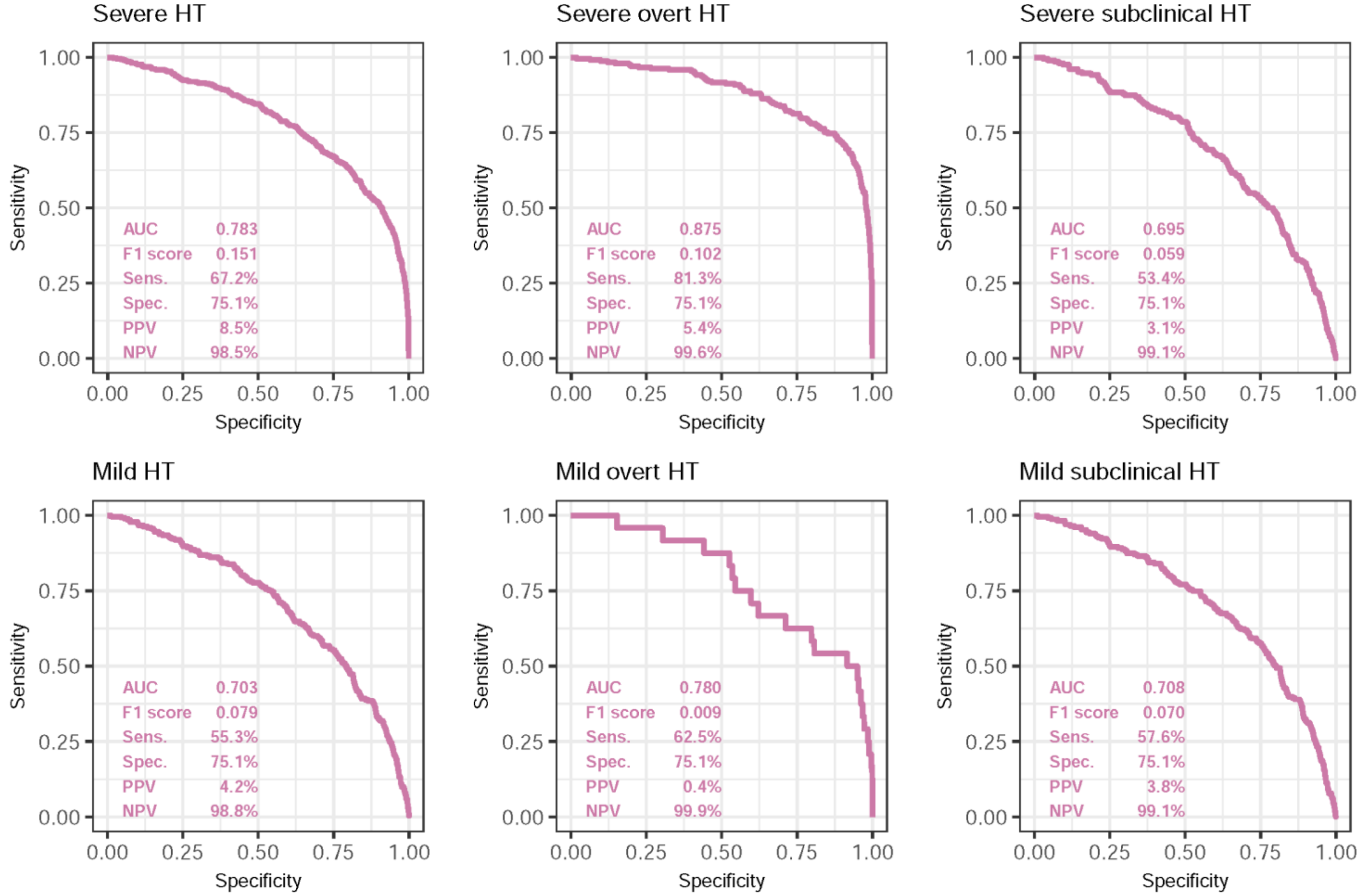

Supplementary Figure 2 | The ROC curve of DLM predictions based on ECG to detect hyperthyroidism (HT), overt HT, and subclinical HT stratified by severe (TSH ≤0.05 μIU/mL) and mild (TSH 0.06-0.50 μIU/mL) condition. The overt HT was defined as a free T4 of ≥1.78 ng/dL, and the ECGs without corresponding free T4 test were excluded. The operating point was selected based on the Figure 3, and the area under ROC curve (AUC), F1 score, sensitivity (Sens.), specificity (Spec.), positive predictive value (PPV), and negative predictive value (NPV) were calculated based on it.

Community test set

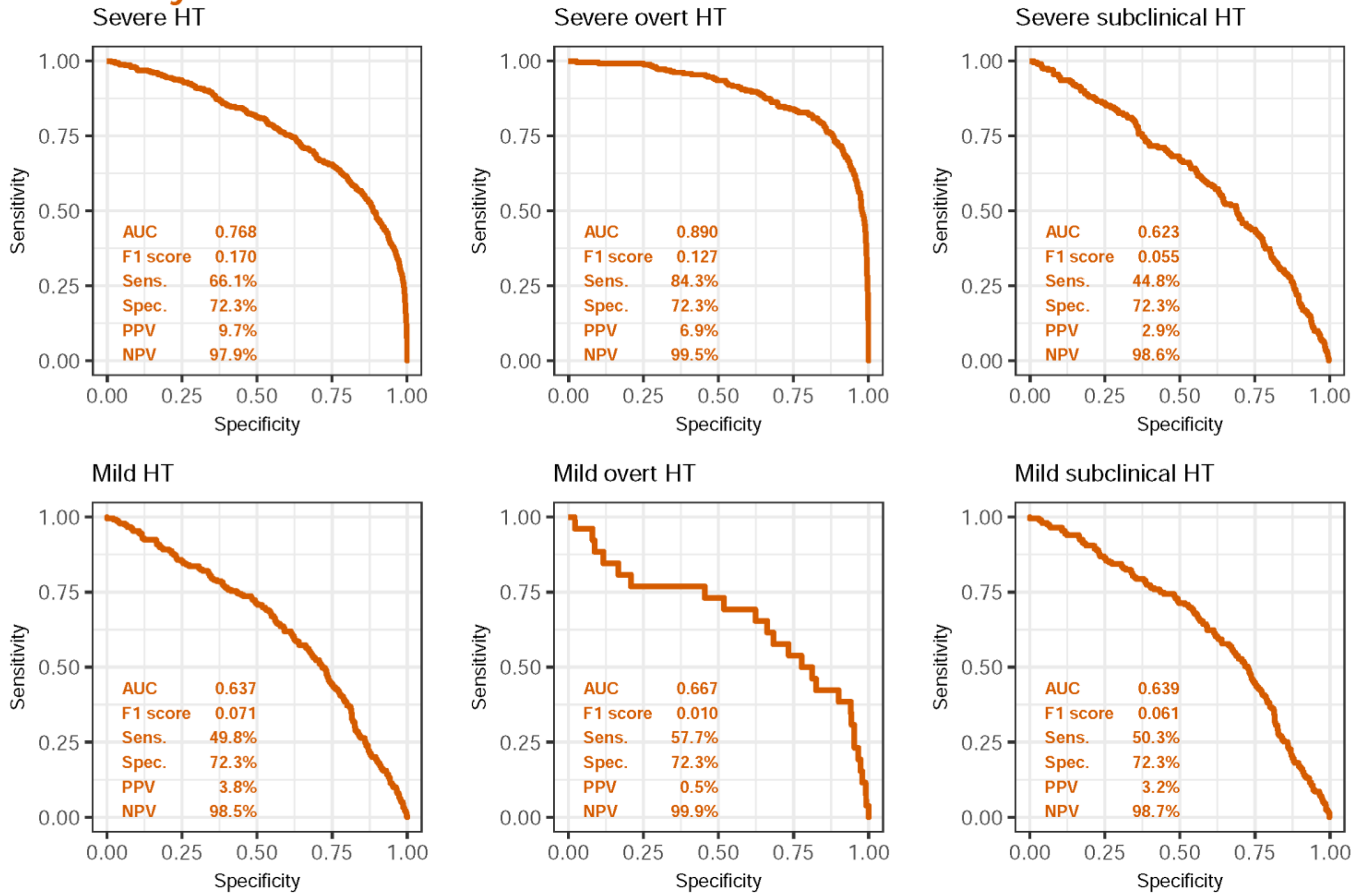

Supplementary Figure 2 (cont.) | The ROC curve of DLM predictions based on ECG to detect hyperthyroidism (HT), overt HT, and subclinical HT stratified by severe (TSH ≤0.05 μIU/mL) and mild (TSH 0.06-0.50 μIU/mL) condition. The overt HT was defined as a free T4 of ≥1.78 ng/dL, and the ECGs without corresponding free T4 test were excluded. The operating point was selected based on the Figure 3, and the area under ROC curve (AUC), F1 score, sensitivity (Sens.), specificity (Spec.), positive predictive value (PPV), and negative predictive value (NPV) were calculated based on it.

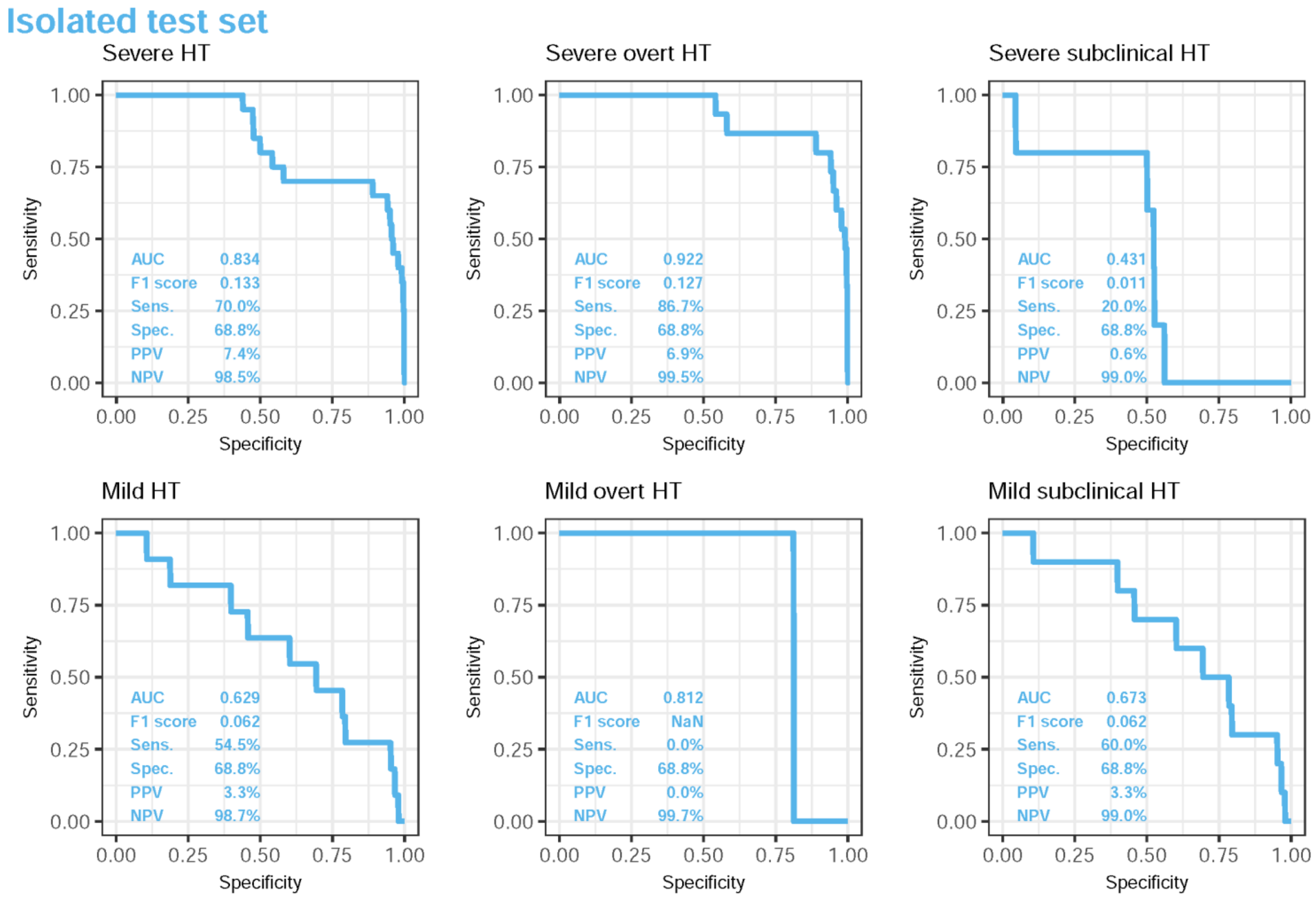

Supplementary Figure 2 (cont.) | The ROC curve of DLM predictions based on ECG to detect hyperthyroidism (HT), overt HT, and subclinical HT stratified by severe (TSH ≤0.05 μIU/mL) and mild (TSH 0.06-0.50 μIU/mL) condition. The overt HT was defined as a free T4 of ≥1.78 ng/dL, and the ECGs without corresponding free T4 test were excluded. The operating point was selected based on the Figure 3, and the area under ROC curve (AUC), F1 score, sensitivity (Sens.), specificity (Spec.), positive predictive value (PPV), and negative predictive value (NPV) were calculated based on it.

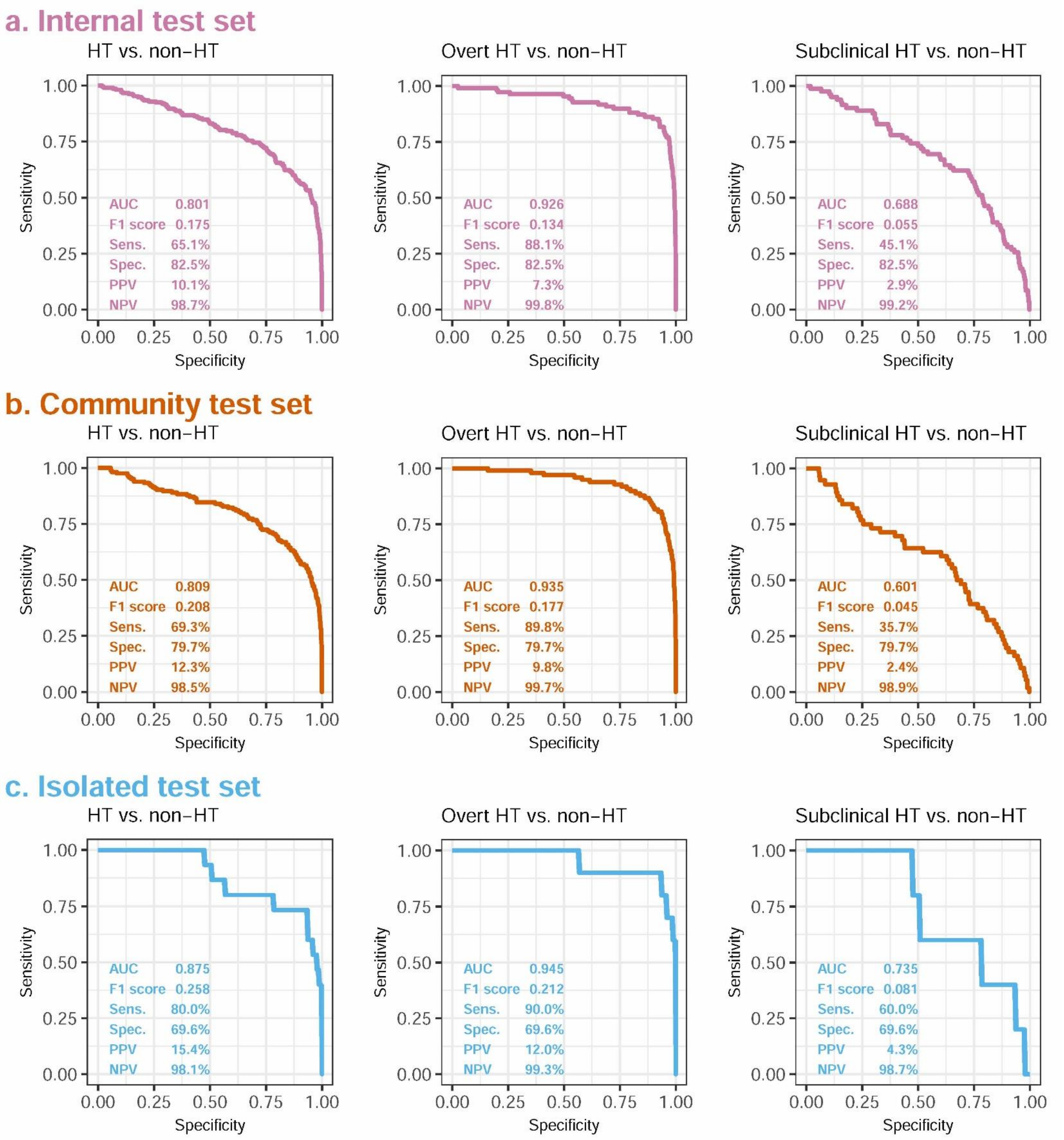

Supplementary Figure 3 | The ROC curve of DLM predictions based on ECG to detect hyperthyroidism (HT), overt HT, and subclinical HT in patients less than 60 years old without histories of HT/anti-thyroid drug and with TSH-ECG pair within 1 days. The overt HT was defined as a free T4 of ≥1.78 ng/dL, and the ECGs without corresponding free T4 test were excluded. The operating point was selected based on the Figure 3, and the area under ROC curve (AUC), F1 score, sensitivity (Sens.), specificity (Spec.), positive predictive value (PPV), and negative predictive value (NPV) were calculated based on it. We presented the performance in internal test set (a), community test set (b), and isolated test set (c), respectively.

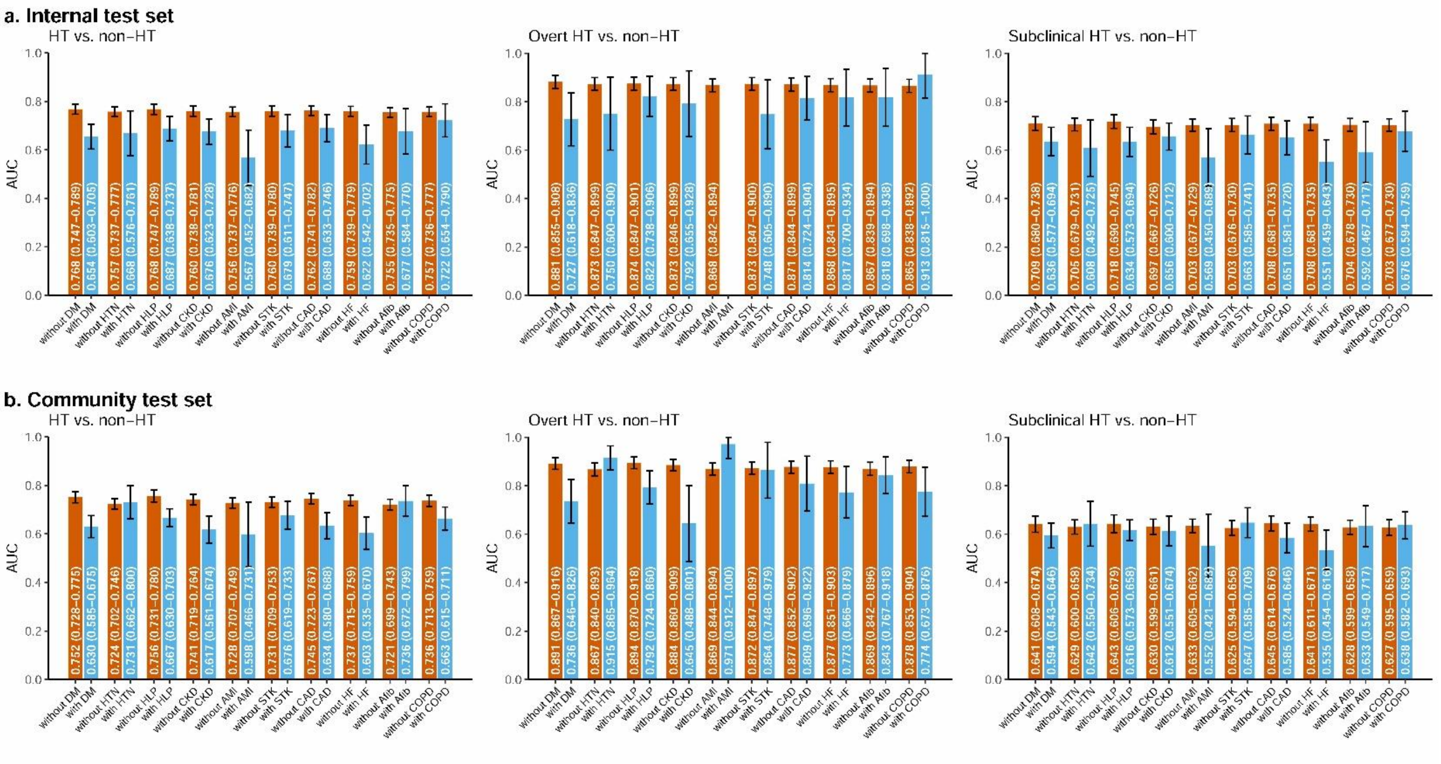

Supplementary Figure 4 | Stratified analysis for AI-ECG performance for predicting hyperthyroidism (HT), overt HT, and subclinical HT. The analyses were stratified by the disease histories. The area under curve (AUC) and 95% confident intervals (CI) were presented based on mild to severe abnormal cardiac functions. We presented the performance in internal test set (a) and, community test set (b), respectively.
