# Supplementary material for: Artificial intelligence-enabled electrocardiography contributes to hyperthyroidism detection and outcome prediction
Source: Commun Med (Lond). 2024 Mar 12;4:42. doi: 10.1038/s43856-024-00472-4 (PMC10933352; doi:10.1038/s43856-024-00472-4)
Supplement: Supplementary file 2 — Reporting summary [file 43856_2024_472_MOESM2_ESM.pdf]

nature portfolio

Corresponding author(s): Shih-Hua Lin
Last updated by author(s): Mar 30, 2023

Reporting Summary

Nature Portfolio wishes to improve the reproducibility of the work that we publish. This form provides structure for consistency and transparency in reporting. For further information on Nature Portfolio policies, see our Editorial Policies and the Editorial Policy Checklist.

Statistics

For all statistical analyses, confirm that the following items are present in the figure legend, table legend, main text, or Methods section.

n/a	Confirmed	
☐	☒	The exact sample size (n) for each experimental group/condition, given as a discrete number and unit of measurement
☐	☒	A statement on whether measurements were taken from distinct samples or whether the same sample was measured repeatedly
☐	☒	The statistical test(s) used AND whether they are one- or two-sided<br>*Only common tests should be described solely by name; describe more complex techniques in the Methods section.*
☐	☒	A description of all covariates tested
☒	☐	A description of any assumptions or corrections, such as tests of normality and adjustment for multiple comparisons
☐	☒	A full description of the statistical parameters including central tendency (e.g. means) or other basic estimates (e.g. regression coefficient) AND variation (e.g. standard deviation) or associated estimates of uncertainty (e.g. confidence intervals)
☐	☒	For null hypothesis testing, the test statistic (e.g. F, t, r) with confidence intervals, effect sizes, degrees of freedom and P value noted<br>*Give P values as exact values whenever suitable.*
☒	☐	For Bayesian analysis, information on the choice of priors and Markov chain Monte Carlo settings
☒	☐	For hierarchical and complex designs, identification of the appropriate level for tests and full reporting of outcomes
☒	☐	Estimates of effect sizes (e.g. Cohen's d, Pearson's r), indicating how they were calculated

Our web collection on statistics for biologists contains articles on many of the points above.

Software and code

Policy information about availability of computer code

Data collection	All data utilized in this study were electronically retrieved. R, FTP, SQL and other core systems of our data warehouse were utilized.
Data analysis	Data analysis R version 3.4.4; Model training and inference R version 3.4.4 and Mxnet version 1.3.0.

For manuscripts utilizing custom algorithms or software that are central to the research but not yet described in published literature, software must be made available to editors and reviewers. We strongly encourage code deposition in a community repository (e.g. GitHub). See the Nature Portfolio guidelines for submitting code & software for further information.

Data

Policy information about availability of data

All manuscripts must include a data availability statement. This statement should provide the following information, where applicable:

- Accession codes, unique identifiers, or web links for publicly available datasets
- A description of any restrictions on data availability
- For clinical datasets or third party data, please ensure that the statement adheres to our policy

The data analyzed in this study is not publicly available due to privacy and security concerns. The data may be shared with a third party upon execution of data sharing agreement for reasonable requests, such requests should be addressed to C. Lin or S.H. Lin.

Field-specific reporting

Please select the one below that is the best fit for your research. If you are not sure, read the appropriate sections before making your selection.

☒ Life sciences ☐ Behavioural & social sciences ☐ Ecological, evolutionary & environmental sciences

For a reference copy of the document with all sections, see nature.com/documents/nr-reporting-summary-flat.pdf

Life sciences study design

All studies must disclose on these points even when the disclosure is negative.

Sample size	This study does not follow the traditional null hypothesis testing strategy that lends itself to power calculation (i.e., there is no null hypothesis for the algorithm). Instead, we abstracted all of the cases that met inclusion criteria and utilized measures of statistical precision. For sample sizes over 2,000 (>20,000 validation samples in this study), which is this case for this study, precision of proportions (i.e., AUC, accuracy, sensitivity, etc) have precision <1 percentage point. Thus, the sample size of the study was sufficiently sized to support the research objectives.
Data exclusions	There is no data excluded in this study.
Replication	The study design included a deep learning model based on data from an independent development set. Moreover, two external validation sets from different hospitals were also used to validate its replicaction.
Randomization	This was a retrospective chart review study. There was no intervention or treatment assignment as a result. Therefore, randomization is not applicable.
Blinding	As noted in the randomization section above, there was no randomization or treatment assignment. Accordingly, blinding was not applicable to this study.

Reporting for specific materials, systems and methods

We require information from authors about some types of materials, experimental systems and methods used in many studies. Here, indicate whether each material, system or method listed is relevant to your study. If you are not sure if a list item applies to your research, read the appropriate section before selecting a response.

Materials & experimental systems

n/a	Involved in the study	
☒	☐	Antibodies
☒	☐	Eukaryotic cell lines
☒	☐	Palaeontology and archaeology
☒	☐	Animals and other organisms
☐	☒	Human research participants
☒	☐	Clinical data
☒	☐	Dual use research of concern

Methods

n/a	Involved in the study	
☒	☐	ChIP-seq
☒	☐	Flow cytometry
☒	☐	MRI-based neuroimaging

Human research participants

Policy information about studies involving human research participants

Population characteristics	All 12-lead ECGs recorded on patients who had also received at least 1 serum TSH test within 3 days before or after the ECG were used. Hospitals A (an academic center), B (a community hospital), and C (a local hospital) included 47,666, 11,498, and 596 eligible patients, respectively. The overall patient populations had mean age of 54.2+/-18.4, 58.5+/-19.3, and 59.4+/-17.7 in hospitals A, B, and C, respectively, and 5-6% of the population had hyperthyroidism.
Recruitment	N/A - records based research
Ethics oversight	This study was approved by the institutional review board of Tri-Service General Hospital, Taipei, Taiwan (IRB NO. C202105049). Patients' consent was waived because data were collected retrospectively and in anonymized files and encrypted from the hospital to the data controller.

Note that full information on the approval of the study protocol must also be provided in the manuscript.
